# Isolation and characterization of novel lipases/esterases from a bovine rumen metagenome

**DOI:** 10.1007/s00253-014-6355-6

**Published:** 2015-01-11

**Authors:** Florence Privé, C Jamie Newbold, Naheed N. Kaderbhai, Susan G. Girdwood, Olga V. Golyshina, Peter N. Golyshin, Nigel D. Scollan, Sharon A. Huws

**Affiliations:** 1 Institute of Biological, Environmental and Rural Sciences, Aberystwyth University, Aberystwyth, SY23 3DA UK; 2 School of Biological Sciences, Bangor University, Bangor, LL57 2UW UK

**Keywords:** Rumen, Lipolysis, Fatty acid, Lipase, Esterase, Bacteria, Functional metagenomic

## Abstract

Improving the health beneficial fatty acid content of meat and milk is a major challenge requiring an increased understanding of rumen lipid metabolism. In this study, we isolated and characterized rumen bacterial lipases/esterases using functional metagenomics. Metagenomic libraries were constructed from DNA extracted from strained rumen fluid (SRF), solid-attached bacteria (SAB) and liquid-associated rumen bacteria (LAB), ligated into a fosmid vector and subsequently transformed into an *Escherichia coli* host. Fosmid libraries consisted of 7,744; 8,448; and 7,680 clones with an average insert size of 30 to 35 kbp for SRF, SAB and LAB, respectively. Transformants were screened on spirit blue agar plates containing tributyrin for lipase/esterase activity. Five SAB and four LAB clones exhibited lipolytic activity, and no positive clones were found in the SRF library. Fosmids from positive clones were pyrosequenced and twelve putative lipase/esterase genes and two phospholipase genes retrieved. Although the derived proteins clustered into diverse esterase and lipase families, a degree of novelty was seen, with homology ranging from 40 to 78 % following BlastP searches. Isolated lipases/esterases exhibited activity against mostly short- to medium-chain substrates across a range of temperatures and pH. The function of these novel enzymes recovered in ruminal metabolism needs further investigation, alongside their potential industrial uses.

**Electronic supplementary material:**

The online version of this article (doi:10.1007/s00253-014-6355-6) contains supplementary material, which is available to authorized users.

## Introduction

Rumen lipid metabolism plays a significant role in regulating the overall lipid composition of microbial cells and also of meat and milk produced by ruminants (Harfoot and Hazlewood 1997; Scollan et al. 2006; Lourenço et al. 2010; Shingfield et al. 2013). The lipid content of forage ingested by ruminants ranges from 2 to 10 % of the total dry weight (Harfoot and Hazlewood 1997), which represent 1.5 kg of ingested lipids through forage per day by dairy cattle (Harfoot 1978). Dietary lipids enter the rumen either as triglycerides (neutral lipids) in concentrate-based feeds or as glycolipids or phospholipids (polar lipids) in forages (Harfoot and Hazlewood 1997; Bauman et al. 2003). Other polar lipids, like sulpholipids, are also present as minor components in forage (<5 %) (Harfoot and Hazlewood 1997). Fresh forage is typically composed of approx. 50 % 18:3 n-3, 15 % 18:2 n-6 and 15 % 16:0 with the rest being minor contributions from other fatty acids (Huws et al. 2009; Huws et al. 2012).

Nonetheless, the fatty acid content of meat and milk does not directly correspond to that in their diets, with ruminant products being relatively high in saturated fatty acids. This is due to lipolysis and subsequent biohydrogenation of dietary lipids within the rumen. On entering the rumen, lipids are hydrolyzed by lipases/esterases, which results in the liberation of glycerol and unsaturated and saturated fatty acids. These fatty acids go through microbial biohydrogenation and are transformed to more saturated end products. Indeed, approximately 92 % 18:3 n-3 and 86 % 18:2 n-6 ingested are biohydrogenated in the rumen (Lourenço et al. 2010; Huws et al. 2010; Huws et al. 2011; Shingfield et al. 2013; Huws et al. 2014).

Research on lipid metabolism in the rumen has largely focused on biohydrogenation of polyunsaturated fatty acids; however, there is a dearth of data on microbial lipolysis, the first step in lipid metabolism in the rumen. Lipolysis is a crucial step in rumen lipid metabolism, and its control could subsequently alter the degree of ruminal biohydrogenation. It is known that dietary lipids are predominantly hydrolyzed in the rumen by obligate anaerobic bacteria (Jenkins et al. 2008), and there is little convincing evidence that rumen protozoa and/or fungi are significantly involved in ruminal lipolysis (Harfoot and Hazlewood 1997; Lourenço et al. 2010, Jenkins et al. 2008). However, to date, only six pure cultures of obligately anaerobic, lipolytic bacteria have been isolated from the rumen, including *Anaerovibrio lipolytica* isolated in the 1960s (Hobson and Mann 1961; Henderson 1970; Henderson 1971; Prins et al. 1975; Privé et al. 2013) and other bacteria belonging to the genera *Butyrivibrio*, *Clostridium* and *Propionibacterium* (Jarvis and Moore 2010). Nonetheless, the major hurdle of being able to culture many of the rumen bacteria means that we are potentially missing a wealth of information on rumen bacterial lipolysis.

Since the first published paper detailing functional metagenomic-based techniques for enhanced gene discovery in whole populations, irrespective of the ability to culture (Handelsman 1994), there has been an explosion in its use resulting in the discovery of many novel enzymes. Indeed, many new families of lipases/esterases have been discovered as a result of developments in functional metagenomic technologies (Nagarajan 2012). Nonetheless, functional metagenomic studies have only retrieved a small number of lipolytic enzymes, including two lipases from the rumen of cattle (Liu et al. 2009) and two esterases from the rumen of sheep (Bayer et al. 2010). In both cases, the isolated genes had only low similarities with lipases from other environments and their significance in rumen function is unknown.

Clearly, in order to be able to manipulate rumen lipid metabolism through control of lipolysis, we must first gain a better fundamental understanding of lipolysis. As such, the aims of this study were to increase our library of discovered lipases/esterases from the rumen metagenome and to gain a better understanding of ruminal lipases/esterases, their biochemical characteristics and the microbes that possess them.

## Materials and methods

### Rumen sampling and DNA extraction

Rumen contents were collected from four rumen-fistulated, non-lactating Holstein cows (average weight of 731 kg) housed at Trawsgoed experimental farm (Aberystwyth, Ceredigion, Wales). Samples were retrieved under the authorities of the UK Animal (Scientific Procedures) Act (1986). The animals were fed a diet composed of a mixture of grass silage and straw (75:25) ad libitum, and ∼1 kg of sugar beet nuts was fed at 0700 h daily with constant access to fresh water. Sampling was completed 2 h after concentrate feeding. Strained ruminal fluid (SRF), solid-attached bacteria (SAB) and liquid-associated bacteria (LAB) were harvested as described previously by Huws et al. (2010). Essentially, 2 h after the morning feed, whole rumen fluid (1 L) was taken from each steer. Rumen samples were squeezed through a sieve, with some SRF kept, before the separation of rumen solids from the retained solids. SAB were obtained by washing of rumen solids (500 g) in saline (2 L), to detach all loosely attached microorganisms, and subsequently hand squeezing before stomaching solids in saline for 5 min to remove the attached microorganisms. The liquid fraction was then centrifuged (800*g*, 15 min) to remove eukaryotes, before the supernatant was centrifuged twice (13,000*g*, 25 min) and resuspended in saline. LAB were obtained by performing the low-speed and high-speed centrifugal steps as described on the liquid fraction obtained after initial hand squeezing of the retrieved rumen sample.

### Construction of metagenomic libraries

Metagenomic DNA was extracted from 200 μL of SRF, SAB and LAB using the BIO101 FastDNA® Spin Kit for Soil (Qbiogene, Cambridge, UK) following the supplier’s protocol but with 3 × 30-s bead beating with 1-min intervals on ice. The libraries were constructed using the CopyControl™ pCC1FOS™ vector and the reagents supplied in the CopyControl™ Fosmid Library Production Kit (Epicentre, Cambio Ltd., Cambridge, UK), following the supplier’s recommendations. All clones were picked using a colony picker Genetix QPix2 XT (Genetix Ltd., New Milton, England) and subcultured for 20 h in 384-well plates (Genetix Ltd., New Milton, England) containing Luria Bertani broth (LB) with 12.5 μg/mL chloramphenicol and 20 % glycerol. They were then stored at −80 °C.

### Screening for lipase activity

Selective screening of the clones for lipolytic activity was accomplished using spirit blue agar (Sigma-Aldrich Ltd., Dorset, UK) supplemented with 1 % tributyrin. The media were supplemented with 12.5 μg/mL chloramphenicol for selection and 2 mL/L of CopyControl Fosmid Autoinduction Solution (Epicentre, Cambio Ltd., Cambridge, UK) for high-copy-number induction of the clones. The medium was poured into square plates (22 × 22 cm), and the clones were stamped onto the agar using a 384-pin replicator (Genetix Ltd., New Milton, England). After incubation for 48 h at 37 °C, clones surrounded by a blue precipitate on spirit blue agar were selected. Positive clones were retested for lipase activity with a secondary screening, and their fosmids were extracted using the QIAprep® Spin Miniprep kit (Qiagen, Crawley, UK) following the supplier’s recommendations. The fosmid size was determined after restriction cutting with *Bam*HI and analysis on an agarose gel.

### Discovery of lipase/esterase genes within putatively positive fosmids

Putatively lipase/esterase positive fosmids were sequenced using a high-throughput pyrosequencing GS FLX instrument (454 Life Sciences) at Aberystwyth University, UK. The purified lipase-positive fosmids were fragmented to 600–900-bp fragments by nebulization, undertaken according to Roche recommendations. The sheared fosmids were ligated to molecular barcodes (Multiplex Identifiers (MID), Roche Life Sciences; Table S1) containing short oligonucleotide adaptors “A” and “B”. This was in order to specifically tag each sample in the sequencing run. A standard flowgram format (SFF) file was obtained for each sample, and nucleotide sequence data and phred-like quality scores were extracted. The reads from each of the pooled libraries were identified by their MID tags by the data analysis software gsAssembler v2.5.3 (Roche Life Sciences) after the sequencing run. The assembly was done using the default parameters on gsAssembler. BlastN on NCBI was used to trim the vector sequence from the contigs. The GC content was calculated with BioEdit. The open reading frames (ORFs) were characterized using ORF Finder (available at [http://www.ncbi.nlm.nih.gov/gorf/gorf.html]), BlastN (non-redundant nucleotide collection) and BLASTP (non-redundant protein sequences database) on NCBI.

The theoretical molecular mass and isoelectric point of the deduced amino acid sequences were calculated using the Compute pI/MW tool on the ExPASy proteomics server (available at http://expasy.org/tools/pi_tool.html). Signal sequences for peptide cleavage were analyzed using SignalP 4.0 (Petersen et al. 2011) using the Gram-negative model. Conserved domains in the amino acid sequences were analyzed with Conserved Domain Search on NCBI (Marchler-Bauer et al. 2011) and the Pfam database (version 25.0, available at http://pfam.sanger.ac.uk/). Phylogenetic analysis was conducted by carrying out multiple sequence alignments using the ClustalW online tool (available at http://www.ebi.ac.uk/Tools/msa/clustalw2/) for the protein sequences using default settings. Closely related homologs were identified from the NCBI non-redundant database using BLASTP searches. Sequences with alignment >50 % identities and e-value <1e^−10^ were considered. As the classification of lipolytic enzymes is based on the comparison of their protein sequences (Arpigny and Jaeger 1999; Hausmann and Jaeger 2010), the protein sequences from 50 members representing eight lipolytic families were retrieved from NCBI (Liu et al. 2009). A multiple sequence alignment file was constructed using ClustalW online tool on the Pfam conserved domains (α/β hydrolase fold or esterase/lipase domain). MEGA5 software (Tamura et al. 2011) was used to construct the tree using the neighbour-joining method by following Dayhoff PAM matrix model.

### Expression and purification of recombinant lipases

Primers for the amplification of the lipase genes were designed with FastPCR 6.1 (Kalendar et al. 2009), with and without the N-terminal signal sequence where one could be identified (Table S2). The PCR reaction was set up in a total volume of 25 μL as follows: 2 μL of template (∼100 ng), 1 μL of forward and reverse primers (10 pM), 8.5 μL of molecular water and 12.5 μL of PCR mastermix (ImmoMix™, Bioline UK Ltd., London, UK). Initial activation of the Taq was performed for 10 min at 95 °C, followed by 25 cycles as follows: 95 °C for 30 s, 50 °C for 30 s and 72° for 2 min, followed by a final extension at 72 °C for 8 min and holding of samples at 4 °C. After PCR, the products were verified by electrophoresis on a 1 % agarose gel using a 1-kb ladder. The band of interest was cut out with a sterile razor blade and the DNA eluted using the MinElute Gel Extraction kit (Qiagen, Crawley, UK).

The expression of the lipolytic genes was then undertaken using the pTrcHis TOPO® TA Expression kit (Invitrogen, Carlsbad, CA, USA) following the supplier’s protocol. The PCR product was ligated to the pTrcHis TOPO vector and transformed into *Escherichia coli* TOP10 cells. Twelve colonies for each transformation were picked for secondary screening, and their insert was analyzed for size and orientation by tip-dip PCR using the gene-specific forward primer and the vector-specific pTrcHis reverse primer (5′-GATTTAATCTGTATCAGG-3′). Proteins were purified from 50 mL cultures in the presence of 50 μg/mL ampicillin. A starter culture (2 mL) grown in LB containing 50 μg/mL ampicillin (inoculated with a single colony) was incubated overnight at 37 °C with shaking. The starter culture was used to inoculate 50 mL of LB (2 % inoculation) followed by incubation at 37 °C with shaking for 2 h. Following the 2-h incubation (mid-log growth phase), the culture was induced with 1 mM isopropyl β-d-1-thiogalactopyranoside (IPTG) and incubated at 37 °C with shaking for 5 h to express the enzyme (this was previously validated to be the optimum conditions for maximal protein expression). The cells were then harvested by centrifugation at 3000×*g*, for 10 min, at 4 °C, and the pellets stored at −80 °C before proceeding to protein purification. Purification of the proteins from the whole pellet was carried out in native conditions using the ProBond™ Purification System (Invitrogen, Carlsbad, CA, USA) following the supplier’s protocol. Protein was eluted with 8 mL native elution buffer (pH 8.0) (50 mM monobasic sodium phosphate (pH 8.0), 0.5 M NaCl, 0.25 M imidazole (pH 6.0)). The purity of the proteins was examined by sodium dodecyl sulphate polyacrylamide gel electrophoresis (SDS-PAGE). SDS-PAGE was performed using a 15 % separating and a 4.5 % stacking gel. Protein concentrations were estimated using the Bradford procedure (Bradford, 1976) employing BSA as the standard (Sigma, Dorset, UK).

### Enzymatic assays

Enzyme activity was quantified on a temperature-controlled PowerWave XS microplate reader (BioTek Instruments Inc., Potton, UK) based on the concentration of ρ-nitrophenol released following the hydrolysis of ρ-nitrophenyl ester substrates by the enzyme. The production of ρ-nitrophenol was monitored in triplicate every minute for 10 min at 410 nm, and data were collected with the software Gen5 v1.10 (BioTek Instruments Inc., Potton, UK). Unless otherwise described, enzyme activity was measured by a standard assay at 39 °C, with 1 mM ρ-nitrophenyl ester substrates (C4–C18) in 50 mM morpholineethanesulphonic acid (MES, pH 6.5) containing 1 % acetonitrile. After preincubation for 3 min, the reaction was started by the addition of 2 μL of the eluted fraction of purified enzyme (∼0.4 mg/mL). Blank reactions were performed with every measurement to subtract appropriate values for non-enzymatic hydrolysis of the substrate. One unit of enzyme activity was defined as the amount of activity required to release 1 μmol of ρ-nitrophenol/min from ρ-nitrophenyl ester. The enzymes’ substrate specificity was also tested by titrating the release of free fatty acids from triglycerides as described by Pinsirodom and Parkin (2001). Tributyrin (C4), tricaprylin (C8) and triolein (C18:1) were used. An emulsion containing 500 μL of substrate in 5 mL of MES (pH 6.5) containing 0.5 mg of gum arabic was preincubated for 15 min at 39 °C with stirring. The enzyme (140 μL, ∼50 mg) was added to initiate lipolysis, and the time was set to T0. At 2.5-, 5-, 10- and 15-min reactions, 600 μL of the reaction mixture was sampled and transferred to a tube containing 1.2 mL of 95 % (*v*/*v*) ethanol to stop the reaction. The contents of the tube were then titrated with 0.005 M NaOH until the pH reached 10.0. The blank was set as a tube containing 1.2 mL of 95 % (*v*/*v*) ethanol and 500 μL of substrate. One unit of lipase activity was defined as the amount of activity required to produce 1 μmol of fatty acid per minute.

### Effect of pH on enzyme activity

The effect of pH on the enzymes was examined across the pH range 3.5 to 10.0 using a wide-range pH buffer containing 40 mM each of acetic acid, MES, *N*-(2-hydroxyethyl) piperazine-*N*′-ethanesulphonic acid (HEPES), *N*-[Tris(hydroxymethyl) methyl]-3-aminopropanesulphonic sodium salt (TAPS) and *N*-cyclohexyl-3-aminopropane sulphonic acid (CAPS). The pH was adjusted by adding 1 M HCl or 1 M NaOH as appropriate at 39 °C. The specific activity of the enzyme was determined spectrophotometrically at 348 nm as it is the pH-independent isobestic wavelength of ρ-nitrophenoxide and ρ-nitrophenol (Hotta et al. 2002).

### Effect of temperature on enzyme stability and thermostability/refolding efficiency

The effect of temperature on the enzyme activity was examined across the range 25–70 °C under standard assay conditions. The pH of the MES buffer was adjusted to 6.5 at respective temperatures. The thermostability of the enzymes was analyzed by measuring the residual activity after incubating the enzyme (2 μL in 50 mM MES, pH 6.5) for 1 h at 50, 60 and 70 °C.

### Effect of metal ions on enzyme activity

The effect of metal ions on the activity of the enzymes was investigated by incubating the enzymes with various metal chloride salts (Na^+^, K^+^, NH_4_^+^, Mg^2+^, Ca^2+^, Mn^2+^, Zn^2+^, Co^2+^) at final concentrations of 5 mM in 50 mM MES (pH 6.5) for 30 min at room temperature. The remaining activity was then measured under standard assay conditions.

### Sequence accession numbers

The nucleotide sequences of the genes reported here are available in the GenBank database under accession numbers JX469447 to JX469462. The full fosmid sequences are also available in the GenBank database under the BioSample ID: SAMN03144433.

## Results

### Screening for lipase activity from metagenomic libraries

The metagenomic libraries consisted of a total of 23,872 clones: 7,744 from SRF, 8,448 from SAB and 7,680 from LAB, with a range of insert sizes of 30–35 kbp. The libraries were screened using a spirit blue assay. Five clones from the SAB library and four clones from the LAB library were positive for lipolytic/esterase activity (Fig. S1). There were no positive clones observed from the SRF library. The putatively positive SAB fosmids: SAB5A16, SAB16A18, SAB16E6, SAB18J4 and SAB28M4, contained 31, 19, 31, 16 and 20 kbp of metagenomic material, respectively, whilst the LAB fosmids: LAB4P4, LAB8M16, LAB9D24 and LAB9P23, contained of 28, 39, 33 and 18 kbp metagenomic DNA, respectively.

The protein coding sequences in fosmids SAB5A16, SAB16A18, SAB16E6, SAB18J4, SAB28M4, LAB4P4, LAB8M16 and LAB9P23 were more closely related to *Prevotella ruminicola* 23 and *Bacteroides* species; nonetheless, the matches were often low, and possibly, the matches to these bacteria were due to the fact that they have been genome sequenced and, therefore, dominate the GenBank database (Tables S3, S4, S5, S6, S7, S8, S9 and S11). Coding sequences in fosmid LAB9D24 were most closely related to *Butyrivibrio fibrisolvens*, *Ruminococcus* sp., *Bacteroides* sp. and *Prevotella* sp. (Table S10). Fourteen putative genes showing similarity to known esterase/lipase genes were retrieved and were named lip1 to lip14, and two patatin-like phospholipase genes were also found and named pl1 and pl2 (Table 1). No lipase genes were retrieved from LAB8M16 or LAB9D24, either because of the incomplete assembly of the fosmid sequence due to low sequence coverage or possibly because the blue hue observed between 20 and 24 h in the spirit blue agar plate assay was a false positive.

**Table 1:** Putative lipase/esterase genes and features of the encoded proteins identified using a tributyrin spirit blue screen of the rumen metagenome of cattle

Fosmid	Gene	Length (bp)	Protein size (aa)	Protein molecular weight (kDa)	Theoretical isoelectric point
SAB5A16	lip1	1578	525	58.86	4.82
	lip2	1584	527	58.88	4.89
	lip3	1749	582	65.67	4.63
SAB16A18	lip4	1743	580	65.46	4.53
	lip5	1581	526	58.71	4.93
	lip6	1563	520	58.28	4.95
SAB16E6	lip7	1059	352	38.93	5.66
	lip8	930	309	31.79	5.71
	pl1	1239	412	47.38	8.67
SAB18J4	lip9	1560	519	58.26	5.15
	lip10	1677	558	62.58	5.32
SAB28M4	lip11	963	320	35.48	6.41
	lip12	1086	361	40.07	6.34
	pl2	2301	766	85.67	8.77
LAB4P4	lip13	846	281	31.67	6.26
LAB9P23	lip14	1059	352	38.55	5.14

### Classification of lipases

BlastN analysis showed that the gene sequences of lip1, lip2, lip3, lip4, lip5, lip6, lip9 and lip10 had 74 to 80 % sequence identity to the gene o23 coding for an ester hydrolase in an uncultured marine prokaryote (AJ811969). Genes lip7, lip12 and lip14 possessed 70–72 % sequence identity to an esterase gene isolated from an uncultured bacterium from a cow rumen metagenome, whilst lip8 and lip11 possessed 75 % sequence identity to an esterase/lipase gene retrieved from a phagemid clone from a bovine rumen metagenomic library (Table S12). Phospholipase genes pl1 and pl2 did not show any similarity to sequences documented in GenBank using BlastN. The deduced amino acid sequences of the genes were used to perform a BLASTP search against the NCBI database. Lip3, lip4, lip7, lip8, lip11, lip12 and lip14 possessed between 50 and 78 % identity to other esterases/lipases from uncultured rumen bacteria (ADE28720, ABI17943, CAJ19128). A higher similarity (63 to 84 % identity) was also observed between lip1, lip2, lip5, lip6, lip9 and lip10 and the ester hydrolase from an uncultured marine prokaryote (CAH19079). Lip13 possessed 78 % similarity to a lipase from *P. ruminicola* 23. Phospholipases pl1 and pl2 were 40 and 51 % identity, respectively, related to the patatin family phospholipase of *Prevotella oralis* ATCC33269 (Table S13). Nonetheless, despite some similarity to known lipases/esterases, the % homologies confirm that the enzymes discovered within this study have a degree of novelty.

**Fig. 1:**
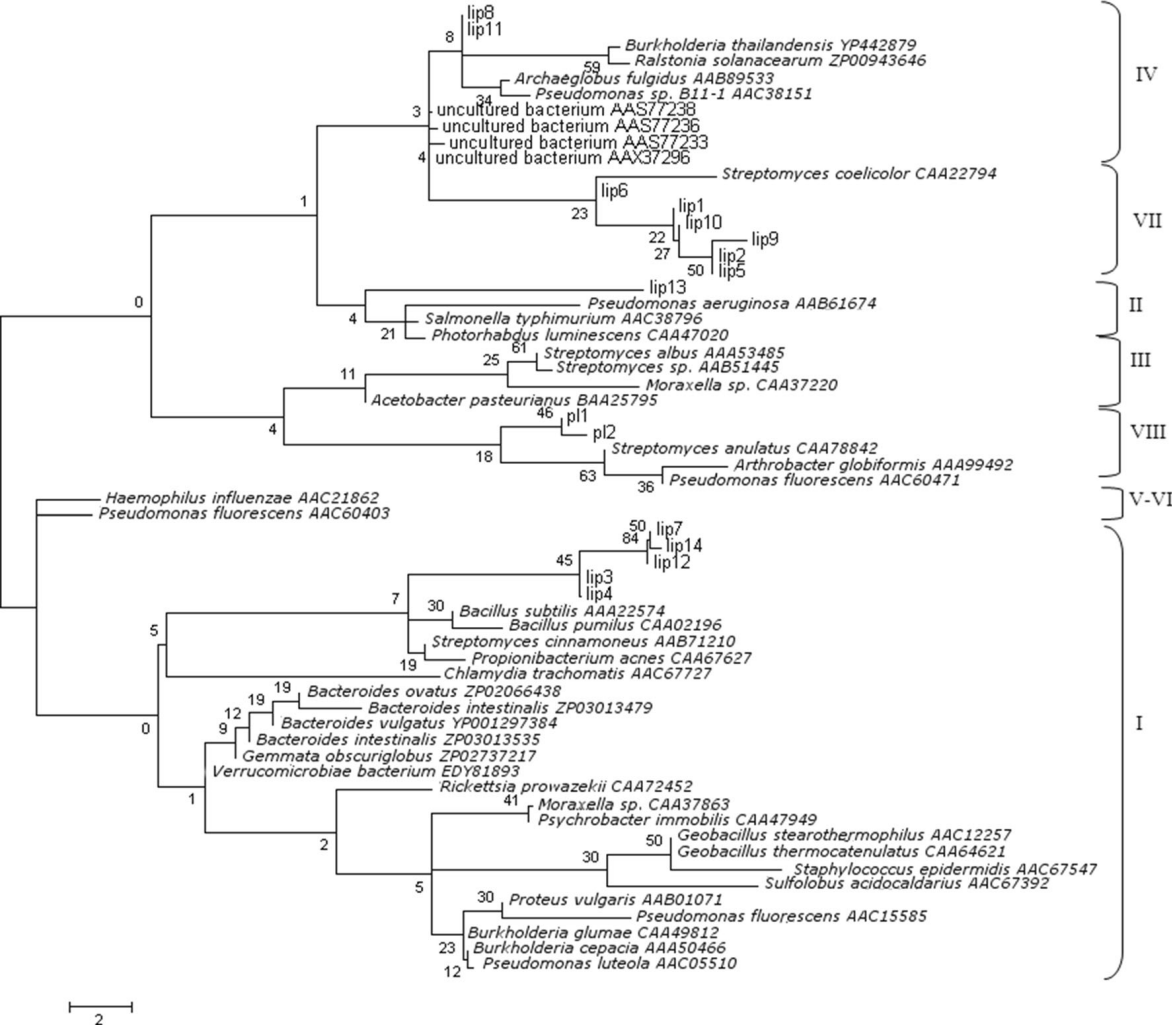
Neighbour-joining analysis of lip1 to lip14 and lipolytic proteins from different families. The scale indicates the number of substitution events. The *numbers associated with the branches* refer to the bootstrap values (confidence limits) resulting from 1000 replicate resamplings. *Roman numerals* correspond to the lipolytic families as defined by Arpigny and Jaeger (1999)

Phylogenetic placement of the predicted proteins suggests lipases from all the main lipase families described by Arpigny and Jaeger (1999) (Fig. 1). Domain analysis confirmed that lip1, lip2, lip5, lip6, lip9 and lip10 contained domains linked to lipase and esterase activity (esterase/lipase superfamily domain, carboxylesterase domain). Lip8 and lip11 contained an α/β hydrolase fold domain, whilst lip3, lip4, lip7, lip12 and lip14 had a DUF3089 domain, which represents an α/β hydrolase fold and, therefore, putative enzymatic activity. Lip13 contained a rhamnogalacturonan esterase-like domain. Pl1 and pl2 were predicted to be outer membrane proteins, and pl2 also contained a patatin-like phospholipase domain and a domain predicted to code for an esterase of the α/β hydrolase family. Proteins lip3, lip4, lip7, lip10, lip12, lip13, lip14 and pl2 were predicted to be secreted enzymes based on the presence of a putative signal peptide.

Lip8 and lip11 clustered with genes from family IV as defined by Arpigny and Jaeger (1999) (Fig. 1). This was confirmed by multiple alignments with proteins from this family (Fig. S2), and lip8 and lip11 contained the lipase-conserved catalytic triad of residues Glu, replacing Asp (residue 229 and 263 for lip8 and lip11, respectively), His (259 and 293, respectively) and the catalytic nucleophile Ser (138, 172), respectively, in the consensus pentapeptide GDSAG. The HSL family conserved HGGG motif, amino acids 69 to 71 and 103 to 105, respectively, was found upstream of the active-site conserved motif. Alignments indicated that lip1, lip2, lip5, lip6, lip9 and lip10 might be more closely related to family VII. Multiple amino acid alignment (Fig. S3) with enzymes related to this family provided confirmation: the catalytic triad was present with Asp, His and Ser in the consensus motif GESAG. Lip13 clustered with the so-called GDSL enzymes from family II. The active site motif GDS(L) was found in the N-terminus of the protein sequence, and elements of the five blocks of conserved amino acids were present in its sequence (Fig. S4). The dendrogram (Fig. 1) suggested that lip3, lip4, lip7, lip12 and lip14 clustered with true lipases from subfamily I.7. Multiple amino acid alignments with lipases included in this family showed that the proteins contained the conserved motif GHSQG (Fig. S5). However, the alignments did not show other conserved blocks and the putative catalytic triad was not identified for lip3, lip4 and lip14 with the Asp missing. Pl1 and pl2 clustered with enzymes from family VIII; however, multiple amino acid alignments did not show conserved motifs characteristic from this enzyme family (data not shown).

### Biochemical characterization of purified lipases/esterases

In order to investigate the biochemical properties of the enzymes, they were expressed using the pTrcHis TOPO vector in *E. coli* TOP10. Based on the level of expression obtained with each protein and in order to analyze proteins from each lipase family (Fig. 1), the proteins from fosmid libraries lip4, lip6, lip13ss, pl1 and pl2ss were chosen for purification and further characterization. Proteins lip13ss and pl2ss were produced using primers that amplified the protein minus signal peptides as the cloning of active lipases with their signal peptides was unsuccessful. Purification, following induction with IPTG (1 mM) for 5 h and after elution from the nickel resin routinely, yielded 0.2 to 0.4 mg/mL of purified protein from 50 mL cultures.

In terms of substrate specificity, Lip4 and lip13ss showed narrow chain length specificity, with the highest specific activity against ρ-nitrophenyl laurate (373.4 and 398.6 U/mg, respectively) and a lower specific activity against ρ-nitrophenyl caprate (107.7 and 214.6 U/mg, respectively); activity against other substrates was very low or not detected (Table 2). Protein lip6 exhibited the typical behaviour for carboxylesterases, showing a preference for short acyl chains (Table 2). The highest specific activity was observed with ρ-nitrophenyl butyrate (273.3 U/mg), and activity values decreased with the increase in the acyl chain length (Table 2). Pl1 showed a broader range of activity with higher specific activities against short to medium acyl chain length: the activities were respectively 247.8 U/mg with ρ-nitrophenyl butyrate, 317.5 U/mg with ρ-nitrophenyl caprylate and 224.6 U/mg with ρ-nitrophenyl caprate and laurate. Pl2ss showed no identifiable substrate preference (Table 2). There was some release of free fatty acids from tributyrin, tricaprylin and triolein indicating some activity against the longer-chain triglycerides (Table 2).

**Table 2:** Substrate specificity of lipases/esterases isolated from the rumen metagenome of cattle

Substrate	Specific activity (U/mg protein)				
	lip4	lip6	lip13ss	pl1	pl2ss
pNP-acyl esters					
Butyrate (C4)	56.3 ± 12.1	273.3 ± 22.5	ND	247.8 ± 11.1	172.5 ± 12.0
Caproate (C6)	28.7 ± 24.9	198.6 ± 10.8	51.1 ± 14.5	154.9 ± 21.8	58.8 ± 20.4
Caprylate (C8)	36.0 ± 23.7	42.4 ± 16.7	20.5 ± 14.4	317.5 ± 31.6	141.2 ± 17.0
Caprate (C10)	107.7 ± 37.3	30.5 ± 5.1	214.6 ± 14.5	224.6 ± 5.5	109.8 ± 4.5
Laurate (C12)	373.4 ± 45.7	23.8 ± 8.7	398.6 ± 7.1	224.6 ± 11.0	274.5 ± 36.3
Myristate (C14)	71.7 ± 12.4	18.7 ± 4.3	153.3 ± 25.6	209.1 ± 20.4	227.4 ± 27.2
Palmitate (C16)	ND	13.6 ± 7.0	ND	162.6 ± 32.4	235.3 ± 67.9
Stearate (C18)	ND	ND	ND	46.5 ± 32.8	109.8 ± 29.7
Triglycerides					
Tributyrin (C4)	55.8	51.6	26.5	130.0	65.8
Tricaprylin (C8)	55.8	56.8	26.5	65.0	131.7
Triolein (C18:1)	55.8	ND	ND	ND	131.7

*ND* not detected

**Fig. 2:**
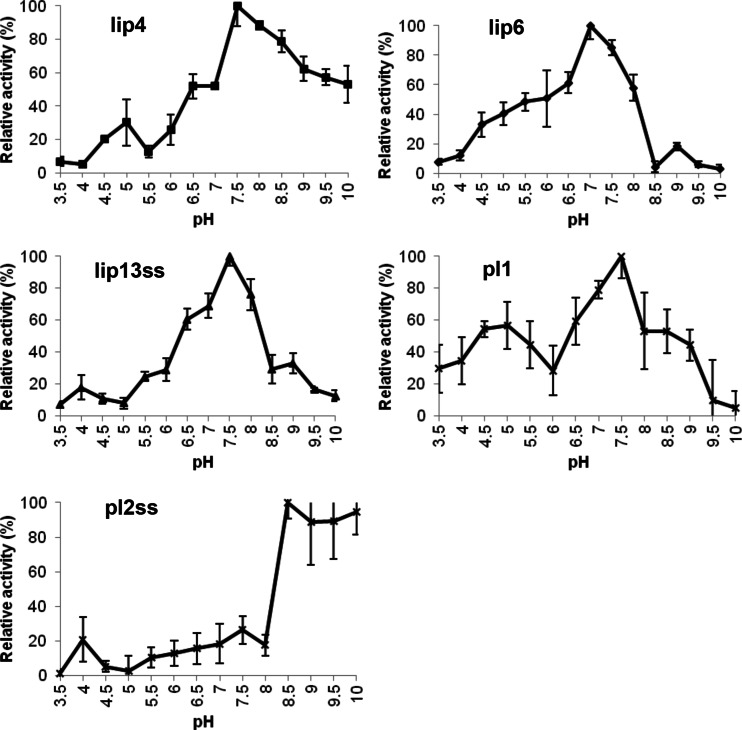
The effect of pH on the activity of lipases isolated from the rumen metagenome of cattle. The pH assays were carried out using ρ-nitrophenyl caprate (C10) as the substrate for lip4 and lip13ss, ρ-nitrophenyl caproate (C6) for lip6 and ρ-nitrophenyl caprylate (C8) for pl1 and pl2ss, at a constant temperature of 39 °C in a wide-range pH buffer set at the indicated pH values

In terms of pH tolerance, proteins Lip4, lip6, lip13ss and pl1 had maximal activity at neutral or slightly alkaline pH (7 or 7.5). Lip6, lip13ss and pl1 exhibited >50 % activity in the pH range of 6.5–8.0, while lip4 showed activity over a broader pH range as it presented 53 % of its maximum activity level at pH 10. Pl2ss had optimum pH at 8.5, respectively, and presented activity >50 % in alkaline pH range 8.5–10.0 (Fig. 2).

**Fig. 3:**
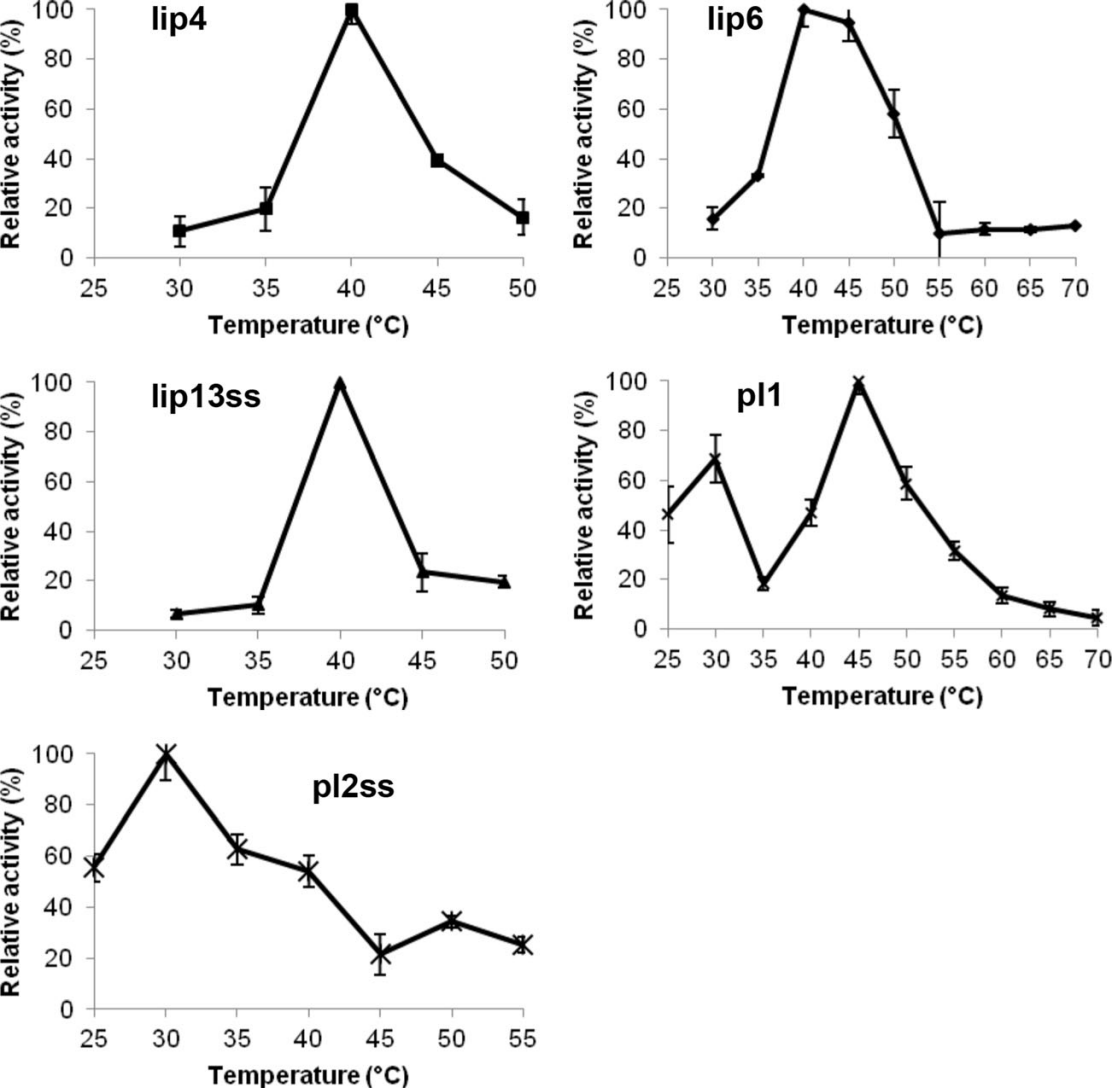
The effect of temperature on the activity of lipases isolated from the rumen metagenome of cattle. The temperature assays were carried out using ρ-nitrophenyl caprate (C10) as the substrate for lip4 and lip13ss, ρ-nitrophenyl caproate (C6) for lip6 and ρ-nitrophenyl caprylate (C8) for pl1 and pl2ss, in a wide-range pH buffer with pH being 6.5 for all assays

The optimum temperatures were determined as 40 °C (lip4, lip6, lip13ss), 45 °C (pl1) and 30 °C (pl2ss) (Table 3 and Fig. 3). The temperature range where the enzyme retained more than 50 % activity was narrow for lip4 (around 40 °C), pl1 (45–50 °C) and lip13ss (around 40 °C), while it was broader for lip6 (40–50 °C) and pl2ss (25–40 °C) (Table 3 and Fig. 3). The proteins lip4, lip13ss, and pl1 appeared to be temperature sensitive as less than 50 % of activity was measured after 1-h incubation at 50 °C (Table 3 and Fig. 3). Lip6 appeared to have some thermostability: at 50 °C, it had 57.9 % activity but lost activity after incubation at 60 or 70 °C (Table 3 and Fig. 3). Protein pl2ss displayed some thermostability as it displayed nearly 90 % of its activity after incubation at 50 and 60 °C and lost only 30 % of its activity after incubation at 70 °C (Table 3 and Fig. 3).

**Table 3:** Relative activity of lipases/esterases isolated from a rumen metagenome of cattle after incubation for 1 h at 50, 60 or 70 °C

Temperature of incubation (°C)	Relative activity (%)				
	lip4	lip6	lip13ss	pl1	pl2ss
40	100	100.0	100.0	100.0	100.0
50	41.8 ± 5.6	57.9 ± 4.3	25.7 ± 11.5	37.0 ± 10.5	87.8 ± 10.3
60	47.5 ± 8.1	11.5 ± 7.8	10.5 ± 18.7	15.1 ± 7.0	89.6 ± 13.2
70	31.3 ± 15.5	13.0 ± 4.3	11.4 ± 3.4	16.5 ± 7.0	73.8 ± 10.9

The enzymes were incubated for 1 h at 50, 60 and 70 °C in 50 mM MES buffer (pH 6.5); the residual activities were measured with a standard assay against ρ-nitrophenyl caprate (C10) for lip4 and lip13ss, ρ-nitrophenyl caproate (C6) for lip6 and ρ-nitrophenyl caprylate (C8) for pl1 and pl2ss. The activity of the enzyme at 40 °C was defined as 100 %

In terms of the effects of ion addition, lip4 activity was strongly inhibited by NH_4_^+^, Mg^2+^, Ca^2+^, Zn^2+^ and Co^2+^ and moderately inhibited by Na^+^ and K^+^, but no effect of Mn^2+^ was observed (Table 4). Lip6 activity was totally inhibited by Mn^2+^ and Co^2+^ and strongly inhibited by K^+^, NH_4_^+^ and Mg^2+^ but only moderately inhibited by Ca^2+^ and Zn^2+^ while Na^+^ slightly increased its activity (Table 4). Only Zn^2+^ had a strong inhibitory effect on pl1 activity while Na^+^, K^+^, NH4^+^, Mg^2+^ and Co^2+^ had moderate inhibitory effects and Ca^2+^ and Mn^2+^ had a stimulatory effect (Table 4). Lip13ss activity was strongly inhibited by K^+^, Mg^2+^, Ca^2+^, Zn^2+^ and Co^2+^ and more moderately by Na^+^, NH4^+^ and Mn^2+^ (Table 4). Pl2ss was totally inhibited by Ca^2+^ and strongly inhibited by Co^2+^ (57 %), but its activity increased significantly with other ions (Table 4).

**Table 4:** Effect of metal ions on the relative activity of lipases isolated from a bovine rumen metagenome

Relative activity (%)					
Ions	lip4	lip6	lip13ss	pl1	pl2ss
None	100.0	100.0	100.0	100.0	100.0
Na^+^	69.8 ± 1.8	101.2 ± 2.4	75.9 ± 1.5	76.2 ± 1.9	119.2 ± 23.0
K^+^	88.4 ± 1.8	16.9 ± 0.5	49.8 ± 2.0	76.2 ± 1.5	260.2 ± 30.7
NH_4_^+^	51.2 ± 1.6	50.6 ± 8.4	61.7 ± 1.1	69.8 ± 1.0	151.8 ± 21.4
Mg^2+^	55.8 ± 9.4	61.8 ± 4.3	26.1 ± 1.7	69.8 ± 1.1	137.3 ± 8.7
Ca^2+^	25.6 ± 1.2	75.9 ± 6.0	45.1 ± 6.0	162.6 ± 11.2	0.0 ± 6.9
Mn^2+^	100.0 ± 6.0	8.4 ± 1.2	90.1 ± 1.1	124.1 ± 12.5	115.6 ± 12.5
Zn^2+^	39.5 ± 1.7	84.3 ± 11.9	7.1 ± 2.5	14.8 ± 2.5	368.6 ± 14.5
Co^2+^	32.6 ± 2.1	0.0 ± 10.7	19.0 ± 8.4	70.9 ± 1.3	57.8 ± 1.3

The enzymes were incubated for 30 min in 50 mM MES buffer (pH 6.5) with the metal ions at 5-mM final concentration; the residual activities were measured with a standard assay against ρ-nitrophenyl caprate (C10) for lip4 and lip13ss, ρ-nitrophenyl caproate (C6) for lip6 and ρ-nitrophenyl caprylate (C8) for pl1 and pl2ss

## Discussion

In this study, we have isolated 14 novel lipase/esterase/phospholipase encoding genes from a bovine rumen microbiome. This is, to our knowledge, one of the most comprehensive of studies in terms of lipase/esterase gene retrieval from the rumen microbiota. This study illustrates that the rumen is a rich resource of novel enzymes, many of which remain undiscovered, and each of which could be useful for industrial applications, as well as serving to increase our fundamental understanding of rumen lipid metabolism.

The genes and the deduced proteins retrieved had varied degrees of similarity to genes previously found in typical ruminal bacteria such as *Bacteroides* and *Prevotella* species (27 to 99 % amino acid similarity). *Prevotella* is one of the most predominant bacterial genera found in the rumen, accounting for up to 20 % of the total bacteria found in sheep (Bekele et al. 2010), between 14 and 60 % in dairy cows (Kong et al. 2010; Stevenson and Weimer, 2007) and up to 90 % in steers (Huws et al. 2010; 2013). The publication of the *P. ruminicola* 23 and *Prevotella bryantii* B(1)4 genomes (Purushe et al. 2010) may explain why most of the fosmid sequences were similar to these entries as only limited information on other rumen bacteria is currently deposited.

The putative esterases and lipases identified were diversely distributed within the eight different lipolytic families defined by Arpigny and Jaeger (1999). They did not cluster in the same families as the two lipases Rlip1 and Rlip2 retrieved from a rumen metagenome library by Liu et al. (2009)). Multiple sequence alignments revealed the presence of highly conserved sequence blocks in the different families, particularly the Gly-Xaa-Ser-Xaa-Gly motif and the catalytic triad Ser, Glu/Asp and His. Lip3, lip4, lip7, lip12 and lip14 are proposed as new members of the lipase subfamily I.7. Little information is available on this family, as only three enzymes have been classified in this group, originating from *Streptomyces cinnamoneus*, *Propionibacterium acnes* and *Corynebacterium glutamicum* (Hausmann and Jaeger, 2010). Lip13 was classified in the esterase family II, or so-called GDSL enzymes, as the catalytic serine is retrieved in a Gly-Asp-Ser-(Leu) tetrapeptide rather than the Gly-Xaa-Ser-Xaa-Gly pentapeptide, and this important motif was found very close to the N-terminus, as noted for other GDSL enzymes (Arpigny and Jaeger 1999). As lip13 carried a rhamnogalacturonan esterase domain, rhamnogalacturonans being a group of plant cell wall pectic polysaccharides, it can be hypothesized that the secreted protein might be involved in plant cell wall degradation in the rumen (Kauppinen et al. 1995; Mølgaard et al. 2000). Lip8 and lip11 were included in family IV; three conserved sequence blocks are observed in these proteins. The HGGG motif, involved in the oxyanion hole stabilization, was found, as well as the possible catalytic triad residues. Lip1, lip2, lip5, lip6, lip9 and lip10 were classified, according to their sequence similarity, as members of family VII. Their molecular masses ranged from 58 to 62 kDa, which is close to the average molecular mass (55 kDa) of esterases belonging to family VII (Hausmann and Jaeger 2010). The physiological role of these esterases is unclear; however, they have attracted interest for their use in many industrial processes (Hausmann and Jaeger 2010). The putative phospholipases pl1 and pl2 were both predicted to be outer membrane proteins (amino acid residues 9 to 241 and 357 to 766, respectively). The amino acid sequence of pl2 also matched on the first part of the protein a predicted patatin-like phospholipase domain (amino acid residues 48 to 254) together with an esterase domain (amino acid residues 44 to 329). Patatin-like proteins have been proposed as a new family of lipolytic enzymes present in bacteria, since they do not share many similarities with other families apart from the Gly-Xaa-Ser-Xaa-Gly motif and were found to be related to eukaryotic phospholipases (Banerji and Flieger 2004). Patatin-like phospholipases have been mainly observed in pathogenic bacteria as virulence factors (Banerji and Flieger 2004).

Phospholipases are ubiquitous and diverse enzymes that mediate various cellular functions, such as membrane maintenance. Phospholipids also constitute most of the plant lipids ingested by herbivores. Phospholipases are classified into four major groups (A, B, C, D) based on their enzymatic specificity and the position at which they cleave within the phospholipid (Sitkiewicz et al. 2007). It is interesting to note that pl2ss activity was increased by ∼3.7-fold in the presence of Zn^2+^, since several phospholipases C involved in pathogenic reactions are zinc metalloenzymes, like the α-toxin from *Clostridium perfringens* (Tsutsui et al. 1995) and the phospholipase C from *Listeria monocytogenes* (Vazquez-Boland et al. 1992) and *Bacillus cereus* (Nakamura et al. 1988). However, pl2ss activity was inhibited in the presence of Ca^2+^, though this cation has often been associated with stimulation of activity due to the formation of calcium salts of long-chain fatty acids (Macrae and Hammond 1985). The lipase from *Pseudomonas aeruginosa* 10145 has likewise been observed to be inhibited, in the presence of calcium ions (Finkelstein et al. 1970). *Helicobacter pylori*, as well as other enteric bacteria, harbours phospholipases A on their outer membrane, and these enzymes participate in the modification of the composition of bacterial membranes, possibly to enhance bacterial growth, colonization and/or survival (Istivan and Coloe 2006). The characterization of these enzymes in the current study is not complete enough to determine their role in bacterial metabolism, and more work is required to assess pl1 and pl2ss role in their hosts. The specific activity observed for pl2ss after 1-h incubation at 50, 60 or 70 °C decreased by only 10 to 30 %, suggesting a possible use of pl2 in biotechnological applications. The half-life of the enzyme at higher temperatures should be assayed to further check this potential.

In summary, we have isolated 14 novel lipases/esterases from rumen bacteria. Lipases/esterases play a key role in regulating fatty acid metabolism in the rumen, and control of lipolysis in the rumen could play a vital role limiting biohydrogenation of polyunsaturated fatty acids. Lipases/esterases are also very important enzymes for many biotechnological processes. Thus, further studies will concentrate on the role of these lipases/esterases in ruminal lipolysis as well as investigating their possible usefulness to the biotechnological industries.

## Electronic supplementary material

Below is the link to the electronic supplementary material.

ESM 1 (PDF 2509 kb)

## References

[CR1] Arpigny JL, Jaeger K-E (1999). Bacterial lipolytic enzymes: classification and properties. Biochem J.

[CR2] Banerji S, Flieger A (2004). Patatin-like proteins: a new family of lipolytic enzymes present in bacteria?. Microbiology.

[CR3] Bauman DE, Perfield JW, De Veth MJ, Lock AL (2003) New perspectives on lipid digestion and metabolism in ruminants. Proc Cornell Nutr Conf 175–189

[CR4] Bayer S, Kunert A, Ballschmiter M, Greiner-Stoeffele T (2010). Indication for a new lipolytic enzyme family: isolation and characterization of two esterases from a metagenomic library. J Mol Microbiol Biotechnol.

[CR5] Bekele AZ, Koike S, Kobayashi Y (2010). Genetic diversity and diet specificity of ruminal *Prevotella* revealed by 16S rRNA gene-based analysis. FEMS Microbiol Lett.

[CR6] Finkelstein AE, Strawich ES, Sonnino S (1970). Characterization and partial purification of a lipase from *Pseudomonas aeruginosa*. Biochim Biophys Acta.

[CR7] Handelsman J (1994). Metagenomics: application of genomics to uncultured microorganisms. Microbiol Mol Biol Rev.

[CR8] Harfoot CG (1978). Lipid metabolism in the rumen. Progr Lipid Res.

[CR9] Harfoot CG, Hazlewood GP, Hobson PN, Stewart CS (1997). Lipid metabolism in the rumen. The rumen microbial ecosystem.

[CR10] Hausmann S, Jaeger K-E, Timmis KN (2010). Lipolytic enzymes from bacteria. Handbook of hydrocarbon and lipid microbiology.

[CR11] Henderson C (1970). The lipases produced by *Anaerovibrio lipolytica* in continuous culture. Biochem J.

[CR12] Henderson C (1971). A study of the lipase produced by *Anaerovibrio lipolytica*, a rumen bacterium. J Gen Microbiol.

[CR13] Hobson PN, Mann SO (1961). The isolation of glycerol fermenting and lipolytic bacteria from the rumen of the sheep. J Gen Microbiol.

[CR14] Hotta Y, Ezaki S, Atomi A, Imanaka T (2002). Extremely stable and versatile carboxylesterase from a hyperthermophilic archaeon. Appl Environ Microbiol.

[CR15] Huws SA, Kim EJ, Lee MRF, Kingston-Smith AH, Wallace RJ, Scollan ND (2009). Rumen protozoa are rich in polyunsaturated fatty acids due to the ingestion of chloroplasts. FEMS Microbiol Ecol.

[CR16] Huws SA, Lee MRF, Muetzel SM, Scott MB, Wallace RJ, Scollan ND (2010). Forage type and fish oil cause shifts in rumen bacterial diversity. FEMS Microbiol Ecol.

[CR17] Huws SA, Kim EJ, Lee MRF, Pinloche E, Wallace RJ, Scollan ND (2011). As yet uncultured bacteria phylogenetically classified as Prevotella, Lachnospiraceae incertae sedis, and unclassified Bacteroidales, Clostridiales and Ruminococcaceae may play a predominant role in ruminal biohydrogenation. Env Micro.

[CR18] Huws SA, Lee MRF, Kingston-Smith AH, Kim EJ, Scott MB, Tweed J, Scollan ND (2012). Ruminal protozoal contribution to the flow of fatty acids following feeding of steers on forages differing in their chloroplast content. British J Nutr.

[CR19] Huws SA, Mayorga OL, Theodorou MK, Kim EJ, Newbold CJ, Kingston-Smith AH (2013). Successional colonisation of perennial ryegrass by rumen bacteria. Lett Appl Microbiol.

[CR20] Huws SA, Kim EJ, Cameron SJS, Girdwood SE, Davies L, Tweed J, Vallin H, Scollan ND (2014). Characterization of the rumen lipidome and microbiome of steers fed a diet supplemented with flax and echium oil. Microb Biotechnol.

[CR21] Istivan TS, Coloe PJ (2006). Phospholipase A in Gram-negative bacteria and its role in pathogenesis. Microbiol.

[CR22] Jarvis GN, Moore ERB (2010) Lipid metabolism and the rumen microbial ecosystem. In: K. N. Timmis (ed) Handbook of hydrocarbon and lipid microbiology. Springer, Berlin Heidelberg, p 2246–2257.

[CR23] Jenkins TC, Wallace RJ, Moate PJ, Mosley EE (2008). Recent advances in biohydrogenation of unsaturated fatty acids within the rumen microbial ecosystem. J Anim Sci.

[CR24] Kalendar R, Lee D, Schulman AH (2009). FastPCR software for PCR primer and probe design and repeat search. Genes, Genomes, Genomics.

[CR25] Kauppinen S, Christgau S, Kofod LV, Halkier T, Dörreich K, Dalbøge H (1995). Molecular cloning and characterization of a rhamnogalacturonan acetylesterase from *Aspergillus aculeatus*. J Biol Chem.

[CR26] Kong Y, Teather R, Forster R (2010). Composition, spatial distribution, and diversity of the bacterial communities in the rumen of cows fed different forages. FEMS Microbiol Ecol.

[CR27] Liu K, Wang J, Bu D, Zhao S, McSweeney C, Yu P, Li D (2009). Isolation and biochemical characterization of two lipases from a metagenomic library of China Holstein cow rumen. Biochem Biophys Res Commun.

[CR28] Lourenço M, Ramos-Morales E, Wallace RJ (2010). The role of microbes in rumen lipolysis and biohydrogenation and their manipulation. Animal.

[CR29] Macrae AR, Hammond RC (1985). Present and future applications of lipases. Biotech Genet Eng Rev.

[CR30] Marchler-Bauer A, Lu S, Anderson JB, Chitsaz F, Derbyshire MK, DeWeese-Scott C, Fong JH, Geer LY, Geer RC, Gonzales NR, Gwadz M, Hurwitz DI, Jackson JD, Ke Z, Lanczycki CJ, Lu F, Marchler GH, Mullokandov M, Omelchenko MV, Robertson CL, Song JS, Thanki N, Yamashita RA, Zhang D, Zhang N, Zheng C, Bryant SH (2011). CDD: a Conserved Domain Database for the functional annotation of proteins. Nucleic Acids Res.

[CR31] Mølgaard A, Kauppinen S, Larsen S (2000). Rhamnogalacturonan acetylesterase elucidates the structure and function of a new family of hydrolases. Structure.

[CR32] Nagarajan S (2012). New tools for exploring “old friends—microbial lipases”. Appl Biochem Biotech.

[CR33] Nakamura S, Yamada A, Tsukagoshi N, Udaka S, Sasaki T, Makino S, Little C, Tomita M, Ikezawa H (1988). Nucleotide sequence and expression in *Escherichia coli* of the gene coding for sphingomyelinase of *Bacillus cereus*. FEBS J.

[CR34] Petersen TN, Brunak S, von Heijne G, Nielsen H (2011). SignalP 4.0: discriminating signal peptides from transmembrane regions. Nat Methods.

[CR35] Prins RA, Lankhorst A, Van der Meer P, Van Nevel CJ (1975). Some characteristics of *Anaerovibrio lipolytica*, a rumen lipolytic organism. Antonie Van Leeuwenhoek.

[CR36] Privé F, Huws SA, Kaderbhai NN, Golyshina OV, Scollan ND, Newbold CJ (2013) Identification and characterization of three novel lipases belonging to families II and V from *Anaerovibrio lipolytica* 5S. PLoS One 8:e69076 10.1371/journal.pone.0069076 PMC3741291 23950883

[CR37] Purushe J, Fouts DE, Morrison M, White BA, Mackie RI.; North American Consortium for Rumen Bacteria, Coutinho PM, Henrissat B, Nelson KE (2010). Comparative genome analysis of *Prevotella ruminicola* and *Prevotella bryantii*: insights into their environmental niche. Microb Ecol.

[CR38] Scollan ND, Hocquette JF, Nuernberg K, Dannenberger D, Richardson I, Moloney A (2006). Innovations in beef production systems that enhance the nutritional and health value of beef lipids and their relationship with meat quality. Meat Sci.

[CR39] Shingfield KJ, Bonnet M, Scollan ND (2013). Recent developments in altering the fatty acid composition of ruminant-derived foods. Animal.

[CR40] Sitkiewicz I, Stockbauer KE, Musser JM (2007). Secreted phospholipase A2 enzymes: better living through phospholipolysis. Trends Microbiol.

[CR41] Stevenson DM, Weimer PJ (2007). Dominance of *Prevotella* and low abundance of classical ruminal bacterial species in the bovine rumen revealed by relative quantification real-time PCR. Appl Microbiol Biotechnol.

[CR42] Tamura K, Peterson D, Peterson N, Stecher G, Nei M, Kumar S (2011). MEGA5: Molecular Evolutionary Genetics Analysis using maximum likelihood, evolutionary distance, and maximum parsimony methods. Mol Biol Evol.

[CR43] Tsutsui K, Minami J, Matsushita O, Katayama S, Taniguch Y, Nakamura S, Nishioka M, Okabe A (1995). Phylogenetic analysis of phospholipase C genes from *Clostridium perfringens* types A to E and *Clostridium novyi*. J Bacteriol.

[CR44] Vazquez-Boland JA, Kocks C, Dramsi S, Ohayon H, Geoffroy C, Mengaud J, Cossart P (1992). Nucleotide sequence of the lecithinase operon of *Listeria monocytogenes* and possible role of lecithinase in cell-to-cell spread. Infect Immun.

