# Supplementary material for: Isolation and characterization of novel lipases/esterases from a bovine rumen metagenome
Source: Appl Microbiol Biotechnol. 2015 Jan 11;99(13):5475–85. doi: 10.1007/s00253-014-6355-6 (PMC4464377; doi:10.1007/s00253-014-6355-6)

Supplementary Material

Journal: Applied Microbiology and Biotechnology

Article Title:Isolation and characterization of novel lipases/esterases from a bovine rumen metagenome

Florence Privé[1], C Jamie Newbold[1], Naheed N. Kaderbhai[1], Susan G. Girdwood[1], Olga V. Golyshina[2], Peter N. Golyshin[2], Nigel D. Scollan[1], Sharon A. Huws[1]

[1]Institute of Biological, Environmental and Rural Sciences, Aberystwyth University, Aberystwyth SY23 3DA, UK

[2]School of Biological Sciences, Bangor University, Bangor LL57 2UW, UK

Corresponding author:

Dr Sharon Huws, IBERS, Aberystwyth University, Aberystwyth SY23 3DA, Ceredigion, UK

Tel: +44 1970 823202

Email address: hnh@aber.ac.uk

Table S1 Mid identifiers used within this study to identify individual fosmid sequences

MID Name	Sequence (5'-3')
MID-1	ACACGACGACT
MID-2	ACACGTAGTAT
MID-3	ACACTACTCGT
MID-4	ACGACACGTAT
MID-5	ACGAGTAGACT
MID-6	ACGCGTCTAGT
MID-7	ACGTACACACT
MID-8	ACGTACTGTGT
MID-9	ACGTAGATCGT

Table S2 Primers used for amplification of the lipolytic genes in the lipase positive fosmid clones

Template	Gene amplified	Primer	Sequence (5'-3')	Expected size of product (bp)
SAB5A16	*lip1*	lip1 F	GATTGGGAGAGAACCTATTTCCTC	1626
		lip1 R	CCGTCAAGGCATATAATAGTTGGT	
	lip2	lip2 F	ACTATGGATGCTCAAGAGATTAGA	1587
		lip2 R	TTAAAGCATATAATATTTGGTGAGGAA	
	lip3	lip3 F	ATGAGACAATTGAAAAAGTGGATGCTT	1773/ 1701(ss)
		lip3 ssF	TCGAACGATGATAATTCCTCC	
		lip3 R	AGCATCCATAGTTTTGTTTCCTTT	
SAB16A18	*lip4*	lip4 F	ATGAGACAATTGAAAAAGTGGATG	1773 / 1710(ss)
		lip4 ssF	GCGTGCTCGTCGAACGAAGAC	
		lip4 R	CTCTTGAGCATCCATAGTTTTGTT	
	lip5	lip5 F	ACTATGGATGCTCAAGAGATTAGA	1620
		lip5 R	CGTATTGTTCCATATTCGTTTTCA	
	lip6	lip5b F	GGAGAGGACCTACTTCCTCAC	1665
		lip5b R	AGCGAGCCCAATTGGTGTGAT	
SAB16E6	*lip7*	lip6 F	AATATGAAAAGACAGAATTTCCTCGTG	1065 / 999(ss)
		lip6 ssF	TCGTGCAAGAGTAGTAACAAA	
		lip6 R	TTCTCACTTTTCCACGAACGC	
	lip8	lip6b F	AGAGTGGCTGCGGTAATCATC	978
		lip6b R	GCCCGACGTAAGTATTCCTAT	
	pl1	pl1 F	CCACTGCGACCGAATGCCATG	1323
		pl1 R	CCTCCGTTGAATCTGTCGATT	
SAB18J4	*lip9*	lip7 F	GGCTGTTCCTGTTACACATAA	1623
		lip7 R	GGTAACTTGAACCTGTCAAAG	
	lip10	lip8 F	ATGAGAAATTTTAAAAAGTGGATGCTTGCC	1695 / 1632(ss)
		lip8 ssF	ACTTCATGCAGTAGCAAAGAAGAC	
		lip8 R	AGTGATCGCGGAAAACAGTCA	
SAB28M4	*lip11*	lip9 F	GCGTTCGTGGAAAAGTGAGAA	1044
		lip9 R	GCCCGACGTAAGTATTCCTAT	
	lip12	lip10 F	TCCGGTTGATGAAGAAATATGCAA	1107 / 999(ss)
		lip10 ssF	TCGTGCAAGAGCAGTAACAAACCC	
		lip10 R	TTCTCACTTTTCCACGAACGC	
	pl2	pl2 F	GACGCCCCTGGAATATAGAATTAA	2340 / 2247(ss)
		pl2 ssF	GCTAAGTCAGAGAAAGAGGCT	
		pl2 R	TTAGAATTCATATCCGAGGTTGAT	
LAB4P4	*lip13*	lip11 F	ATGATGAGAAGTCTGAAGGTT	849 / 789 (ss)
		lip11 ssF	GCCACACCCGACAAGACCACT	
		lip11 R	AACTTATTTTGCGTTCCGAAC	
LAB9P23	*lip14*	lip12 F	GCCAGGCTACTGGACAAGAAT	1101 / 993(ss)
		lip12 ssF	TCGTGCAAGATGAGTAACAAA	
		lip12 R	TTGTTATTTCTCCAGGAATGC	

F, forward primer; **ssF,** forward primer with predicted signal sequence removed; **R,** reverse primer

Table S3 Predicted protein-coding genes in fosmid SAB5A16

ORF	Protein size (aa)	Putative function	Most similar homolog (e-value)	Identity (overlapped aa)
1	609	ATP-dependent DNA helicase RecQ	*Prevotella ruminicola* 23 YP_003573438 (0.0)	532/604 (88%)
2	495	Inosine-5'-monophosphate dehydrogenase	*Prevotella ruminicola* 23 YP_003573439 (0.0)	472/495 (95%)
3	444	Peptidyl-prolyl cis-trans isomerase	*Prevotella ruminicola* 23 YP_003573440 ($4e^{-136}$)	239/450 (53%)
4	451	Survival protein SurA precursor	*Prevotella ruminicola* 23 YP_003573441 (0.0)	357/434 (82%)
5	516	Hypothetical protein	*Prevotella ruminicola* 23 YP_003573442 (0.0)	421/513 (82%)
6	608	DNA mismatch repair protein MutL	*Prevotella ruminicola* 23 YP_003573443 (0.0)	450/613 (73%)
7	780	Beta-xylosidase	*Prevotella ruminicola* 23 YP_003575568 (0.0)	598/772 (77%)
8	759	GTP pyrophosphokinase, (p)ppGpp synthetase II / Guanosine-3',5'-bis(diphosphate) 3'-pyrophospho hydrolase	*Prevotella ruminicola* 23 YP_003573421 (0.0)	634/745 (85%)
9	429	Membrane-bound lytic murein transglycosylase D precursor	*Prevotella ruminicola* 23 YP_003573422 ($9e^{-175}$)	286/378 (76%)
10	187	Hypothetical protein	*Prevotella ruminicola* 23 YP_003573423 ($8e^{-67}$)	114/156 (73%)
11	308	Chromosome (plasmid) partitioning protein ParB / Stage 0 sporulation protein J	*Prevotella ruminicola* 23 YP_003573424 ($3e^{-136}$)	227/307 (74%)
12	257	Chromosome (plasmid) partitioning protein ParA / Sporulation initiation inhibitor protein Soj	*Prevotella ruminicola* 23 YP_003573425 ($9e^{-136}$)	239/255 (94%)
13	248	5-nucleotidase SurE	*Prevotella ruminicola* 23 YP_003573426 ($3e^{-111}$)	186/246 (76%)
14	371	Lipid-A-disaccharide synthase	*Prevotella ruminicola* 23 YP_003573427 ($5e^{-145}$)	250/364 (69%)
15	248	Hypothetical protein	*Prevotella ruminicola* 23 YP_003573428 ($1e^{-91}$)	167/246 (68%)
**16**	**525**	**Thermostable carboxylesterase**	**uncultured prokaryote CAH19079 (0.0)**	**386/515 (75%)**
**17**	**527**	**Carboxylesterase, type B**	**uncultured prokaryote CAH19079 (0.0)**	**326/518 (63%)**
**18**	**582**	**Esterase**	**uncultured bacterium ADE28720 ($8e^{-101}$)**	**187/310 (60%)**
19	360	Hypothetical protein	*Bacteroides cellulosilyticus* ZP_03677910 ($1e^{-17}$)	75/244 (31%)
20	473	Tetratricopeptide repeat family protein	*Prevotella ruminicola* 23 YP_003575201 (0.0)	350/474 (74%)
21	301	Putative phosphate ABC transporter, phosphate-binding component	*Prevotella ruminicola* 23 YP_003575200 ($6e^{-154}$)	256/301 (85%)
22	277	Ferric siderophore transport system, periplasmic binding protein TonB	*Prevotella ruminicola* 23 YP_003575199 ($3e^{-139}$)	254/277 (92%)
23	221	Biopolymer transport protein ExbD/TolR	*Prevotella ruminicola* 23 YP_003575198 ($2e^{-117}$)	205/221 (93%)
24	205	Biopolymer transport protein ExbD/TolR	*Prevotella ruminicola* 23 YP_003575197 ($2e^{-106}$)	184/205 (90%)
25	275	MotA/TolQ/ExbB proton channel family protein	*Prevotella ruminicola* 23 YP_003575196 ($3e^{-139}$)	243/263 (92%)

Table S3 -continued

ORF	Protein size (aa)	Putative function	Most similar homolog (e-value)	Identity (overlapped aa)
26	379	Aspartate aminotransferase	*Prevotella ruminicola* 23 YP_003575195 (0.0)	343/379 (91%)
27	403	3,4-dihydroxy-2-butanone 4-phosphate synthase / GTP cyclohydrolase II	*Prevotella ruminicola* 23 YP_003575194 (0.0)	373/400 (93%)
28	645	Putative membrane protein	*Prevotella ruminicola* 23 YP_003575193 (0.0)	433/638 (68%)
29	134	Hypothetical protein	*Prevotella ruminicola* 23 YP_003575192 ($7e^{-49}$)	104/134 (78%)
30	423	Guanine deaminase	*Clostridium difficile* ATCC 43255 ($2e^{-143}$)	234/422 (55%)

ORFs are numbered following the sense 5’-3’.

Table S4 Predicted protein-coding genes in fosmid SAB16A18

ORF	Protein size (aa)	Putative function	Most similar homolog (e-value)	Identity (overlapped aa)
1	248	YbbM seven transmembrane helix protein	*Prevotella ruminicola* 23 YP_003574730 ($3e^{-114}$)	240/248 (97%)
2	200	Iron(III) dicitrate transport system (permease)/ABC transporter ATP-binding protein	*Prevotella ruminicola* 23 YP_003574729 ($6e^{-94}$)	180/200 (90%)
3	324	Agmatine deiminase	*Prevotella ruminicola* 23 YP_003574728 (0.0)	313/338 (93%)
4	286	N-carbamoylputrescine amidase	*Prevotella ruminicola* 23 YP_003574727 ($2e^{-167}$)	283/286 (99%)
5	298	Hypothetical protein	*Bacteroides cellulosilyticus* DSM 14838 ($5e^{-17}$)	73/244 (30%)
**6**	**580**	**Esterase**	**uncultured bacterium ADE28720 ($1e^{-100}$)**	**187/310 (60%)**
**7**	**526**	**Thermostable carboxylesterase**	**uncultured prokaryote CAH19079 (0.0)**	**327/518 (63%)**
**8**	**520**	**Thermostable carboxylesterase**	**uncultured prokaryote CAH19079 (0.0)**	**387/515 (75%)**
9	166	Hypothetical protein	*Prevotella bryantii* B14 ZP_07059568 ($1e^{-56}$)	109/160 (68%)
10	420	Multi antimicrobial extrusion protein (Na(+)/drug antiporter), MATE family of MDR efflux pumps	*Prevotella* sp. *oral* taxon 472 str. F0295 ZP_05917013 ($5e^{-97}$)	175/393 (45%)
11	56	Low molecular weight protein tyrosine phosphatase	-	-
12	383	Beta-hexosaminidase	*Prevotella ruminicola* 23 YP_003574019 ($2e^{-163}$)	271/378 (72%)
13	221	Hypothetical protein	*Prevotella ruminicola* 23 YP_003574127 ($5e^{-95}$)	157/217 (72%)
14	134	Low molecular weight protein tyrosine phosphatase	*Subdoligranulum variabile* DSM 15176 ($1e^{-56}$)	105/132 (80%)
15	204	D-3-phosphoglycerate dehydrogenase	*Prevotella ruminicola* 23 YP_003574049 ($5e^{-56}$)	103/146 (71%)

ORFs are numbered following the sense 5’-3’. ORFs 1-4 were situated on contig00001, ORFs 5-15 on contig00010.

Table S5 Predicted protein-coding genes in fosmid SAB16E6

ORF	Protein size (aa)	Putative function	Most similar homolog (e-value)	Identity (overlapped aa)
1	259	DNA/RNA endonuclease G	*Prevotella bergensis* DSM 17361 ($5e^{-72}$)	126/236 (53%)
2	384	Endoglucanase C	*Prevotella ruminicola* 23 YP_003575141 (0.0)	333/385 (86%)
3	246	Putative ABC transport system, membrane protein	*Prevotella ruminicola* 23 YP_003574730 ($1e^{-42}$)	98/221 (44%)
4	199	Putative ABC transport system, ATP-binding subunit	*Prevotella ruminicola* 23 YP_003574729 ($5e^{-59}$)	123/199 (62%)
5	336	Agmatine deiminase	*Prevotella ruminicola* 23 YP_003574728 ($1e^{-136}$)	226/338 (67%)
6	291	N-carbamoylputrescine amidase	*Prevotella ruminicola* 23 YP_003574727 ($3e^{-146}$)	243/291 (84%)
7	275	FrrB	*Bacteroides intestinalis* DSM 17393 ($1e^{-34}$)	99/276 (36%)
8	738	Glutamine synthetase type III, GlnN	*Prevotella ruminicola* 23 YP_003574726 (0.0)	639/742 (86%)
9	206	Hypothetical protein	*Prevotella buccae* D17 ZP_06419800 ($1e^{-27}$)	66/178 (37%)
10	135	N-acetylmuramoyl-L-alanine amidase	*Prevotella ruminicola* 23 YP_003574456 ($4e^{-57}$)	101/136 (74%)
11	277	Hypothetical protein (histone-like DNA binding protein)	*Prevotella ruminicola* 23 YP_003574457 ($1e^{-69}$)	121/158 (77%)
12	59	Hypothetical protein	*Prevotella ruminicola* 23 YP_003575129 ($1e^{-05}$)	25/56 (45%)
13	464	Hypothetical protein	*Prevotella ruminicola* 23 YP_003574876 ($4e^{-155}$)	274/462 (59%)
14	112	Hypothetical protein	None	
15	82	Cyclohexadienyl dehydratase	*Prevotella bryantii* B14 ZP_07061441 ($3e^{-31}$)	64/74 (86%)
16	342	Hypothetical protein	*Bacteroides eggerthii* 1_2_48FAA ZP_07933194 ($3e^{-18}$)	90/325 (28%)
17	226	Hypothetical protein	*Prevotella oralis* ATCC 33269 ($4e^{-60}$)	109/221 (49%)
18	335	Hypothetical protein	*Prevotella oralis* ATCC 33269 ($1e^{-104}$)	176/318 (55%)
**19**	**309**	**Esterase/lipase**	**unidentified microorganism CAJ19128 ($1e^{-129}$)**	**223/288 (77%)**
**20**	**352**	**Esterase**	**uncultured bacterium ABI17943 ($5e^{-136}$)**	**232/365 (64%)**
21	404	ABC-type multidrug transport system, permease component	*Prevotella ruminicola* 23 YP_003574570 ($8e^{-141}$)	249/398 (63%)
22	390	ABC-type multidrug transport system, permease component	*Prevotella ruminicola* 23 YP_003574571 ($7e^{-146}$)	241/384 (63%)
23	330	Hypothetical protein	*Prevotella ruminicola* 23 YP_003574572 ($2e^{-130}$)	240/329 (73%)
24	448	Outer membrane efflux protein	*Prevotella ruminicola* 23 YP_003574573 ($6e^{-154}$)	285/445 (64%)
25	57	Hypothetical protein	None	
26	309	Transcriptional regulator, AraC family	*Prevotella ruminicola* 23 YP_003574311 ($3e^{-87}$)	149/307 (49%)
27	633	Serine phosphatase RsbU, regulator of sigma subunit	*Prevotella ruminicola* 23 YP_003574202 ($1e^{-117}$)	251/673 (37%)
28	133	Putative anti sigma factor	*Bacteroides salanitronis* DSM 18170 ($2e^{-33}$)	72/134 (54%)

Table S5 -continued

ORF	Protein size (aa)	Putative function	Most similar homolog (e-value)	Identity (overlapped aa)
29	711	Glycogen debranching enzyme	*Bacteroides salanitronis* DSM 18170 (0.0)	579/705 (91%)
30	91	Putative anti-anti sigma factor	*Bacteroides salanitronis* DSM 18170 ($1e^{-17}$)	42/90 (47%)
31	392	Alpha-amylase	*Entamoeba dispar* SAW760 ($2e^{-45}$)	124/392 (32%)
32	412	Hypothetical protein	*Prevotella oralis* ATCC 33269 ($4e^{-89}$)	167/416 (40%)
**33**	**337**	**Putative patatin-like phospholipase**	***Prevotella oralis* ATCC 33269 ($2e^{-114}$)**	**208/328 (63%)**
34	144	Transcriptional regulator, AraC family	*Prevotella ruminicola* 23 YP_003574311 ($4e^{-43}$)	83/141 (59%)
35	54	Hypothetical protein	None	
36	96	Hypothetical protein	None	
37	78	6-phosphofructokinase	*Prevotella ruminicola* 23 YP_003574519 ($4e^{-29}$)	62/70 (89%)

ORFs are numbered following the sense 5’-3’. ORFs 1-10 were situated on contig00002, ORFs 11-18 on contig00004, ORFs 19-36 on contig00005, ORF 37 on contig 00006.

Table S6 Predicted protein-coding genes in fosmid SAB18J4

ORF	Protein size (aa)	Putative function	Most similar homolog (e-value)	Identity (overlapped aa)
1	184	Hypothetical protein	*Prevotella ruminicola* 23 YP_003574211 ($2e^{-26}$)	61/171 (36%)
2	61	Hypothetical protein	*Prevotella ruminicola* 23 YP_003575067 ($3e^{-04}$)	24/58 (41%)
3	427	Hypothetical protein	*Prevotella copri* DSM 18205 ($8e^{-98}$)	182/405 (45%)
4	173	Hypothetical protein	*Krokinobacter diaphorus* 4H-3-7-5 ($2e^{-09}$)	42/121 (35%)
5	197	Hypothetical protein	None	
6	99	Hypothetical protein	None	
**7**	**519**	**Carboxylesterase type B**	**uncultured prokaryote CAH19079 (0.0)**	**324/517 (63%)**
**8**	**551**	**Carboxylesterase, type B**	**uncultured prokaryote CAH19079 (0.0)**	**433/515 (84%)**
9	1560	Cell surface protein	*Fibrobacter succinogenes* S85 ($2e^{-12}$)	70/200 (35%)
10	201	Hypothetical protein	*Prevotella* sp. *oral* taxon 472 str. F0295 ($2e^{-26}$)	75/199 (38%)
11	61	Hypothetical protein	None	
12	59	Hypothetical protein	None	
13	52	Hypothetical protein	*Prevotella bryantii* B14 ($2e^{-08}$)	28/50 (56%)

ORFs are numbered following the sense 5'-3'. ORFs 1-3 were situated on contig00001, ORF 4 on contig00002, ORFs 5-8 on contig00003, ORFs 9-10 on contig 00004, ORF11 on contig 00009, ORF 12 on contig00018, ORF 13 on contig00020.

Table S7 Predicted protein-coding genes in fosmid SAB28M4

ORF	Protein size (aa)	Putative function	Most similar homolog (e-value)	Identity (overlapped aa)
1	748	Membrane protein, putative	*Prevotella salivae* DSM 15606 (0.0)	504/682 (74%)
2	271	Putative rRNA methylase	*Prevotella ruminicola* 23 YP_003573529 ($7e^{-107}$)	189/260 (73%)
3	218	5'-methylthioadenosine nucleosidase / S-adenosylhomocysteine nucleosidase	*Prevotella ruminicola* 23 YP_0035735298($4e^{-87}$)	157/219 (72%)
4	164	S-ribosylhomocysteine lyase / Autoinducer-2 production protein LuxS	*Prevotella ruminicola* 23 YP_003573527 ($1e^{-82}$)	140/164 (94%)
5	347	Hypothetical protein	*Bacteroides eggerthii* 1_2_48FAA ($6e^{-16}$)	90/337 (27%)
6	764	Hypothetical protein	*Prevotella oralis* ATCC 33269 (0.0)	392/735 (53%)
7	325	6-phosphofructokinase	*Prevotella ruminicola* 23 YP_003574519 ($6e^{-169}$)	292/325 (90%)
**8**	**318**	**Esterase/lipase**	**unidentified microorganism CAJ19128 ($5e^{-140}$)**	**236/310 (76%)**
**9**	**345**	**Esterase**	**uncultured bacterium ABI17943 ($9e^{-134}$)**	**226/356 (63%)**
10	402	ABC-type multidrug transport system, permease component	*Prevotella ruminicola* 23 YP_003574570 ($2e^{-142}$)	253/398 (64%)
11	390	ABC-type multidrug transport system, permease component	*Prevotella ruminicola* 23 YP_003574571 ($2e^{-145}$)	236/384 (61%)
12	330	Hypothetical protein	*Prevotella ruminicola* 23 YP_003574572 ($6e^{-127}$)	237/329 (72%)
13	455	Outer membrane efflux protein	*Prevotella ruminicola* 23 YP_003574573 ($2e^{-162}$)	294/459 (64%)
14	295	Transcriptional regulator, AraC family	*Prevotella ruminicola* 23 YP_003574311 ($2e^{-68}$)	121/247 (49%)
15	646	Serine phosphatase RsbU, regulator of sigma subunit	*Prevotella ruminicola* 23 YP_003574202 ($4e^{-121}$)	258/675 (38%)
16	133	RsbW protein, putative	*Bacteroides salanitronis* DSM 18170 ($3e^{-32}$)	69/134 (51%)
17	701	Glycogen debranching enzyme	*Bacteroides salanitronis* DSM 18170 (0.0)	575/705 (82%)
18	91	Putative anti-anti sigma factor	*Bacteroides salanitronis* DSM 18170 ($8e^{-18}$)	44/90 (49%)
19	400	Neopullulanase	*Entamoeba dispar* SAW760 ($3e^{-41}$)	120/407 (29%)
**20**	**766**	**Putative patatin-like phospholipase**	***Prevotella oralis* ATCC 33269 (0.0)**	**371/732 (51%)**
21	303	Transcriptional regulator, AraC family	*Prevotella ruminicola* 23 YP_003574311 ($6e^{-93}$)	159/298 (53%)
22	590	Hypothetical protein	*Paraprevotella xylaniphila* YIT 11841 ($7e^{-67}$)	201/602 (33%)
23	913	TonB-dependent receptor	*Capnocytophaga* sp. *oral* taxon 329 str. F0087 (0.0)	439/902 (49%)

ORFs are numbered following the sense 5'-3'.

Table S8 Predicted protein-coding genes in fosmid LAB4P4

ORF	Protein size (aa)	Putative function	Most similar homolog (e-value)	Identity (overlapped aa)
1	65	Hypothetical protein	*Prevotella ruminicola* 23 YP_003574638 ($8e^{-19}$)	44/61 (72%)
2	203	Hypothetical protein	*Bacteroides helcogenes* P36-108 ($1e^{-05}$)	48/165 (48%)
3	99	Hypothetical protein	None	
4	318	Beta-galactosidase	*Bacteroides* sp. 20_3 ($1e^{-96}$)	170/292 (58%)
5	99	FIG01162590: hypothetical protein	*Bacteroides caccae* ATCC 43185 ($2e^{-35}$)	69/99 (70%)
6	173	Hypothetical protein	*Prevotella ruminicola* 23 YP_003574211 ($2e^{-27}$)	
7	59	Hypothetical protein	None	
8	50	Hypothetical protein	*Prevotella ruminicola* 23 YP_003574078 ($5e^{-10}$)	33/46 (72%)
9	159	Hypothetical protein	*Bacteroides helcogenes* P36-108 ($3e^{-14}$)	54/154 (35%)
10	69	Hypothetical protein	*Prevotella ruminicola* 23 YP_003574299 ($4e^{-21}$)	50/66 (85%)
11	114	Plasmid maintenance system antidote protein	*Prevotella ruminicola* 23 YP_003574300 ($7e^{-39}$)	80/94 (85%)
12	60	Hypothetical protein	None	
13	132	Hypothetical protein	None	
14	157	Hypothetical protein	*Prevotella oris* F0302 ($3e^{-04}$)	27/88 (31%)
15	123	Hypothetical protein	*Prevotella ruminicola* 23 YP_003574334 ($4e^{-05}$)	31/110 (28%)
16	97	Hypothetical protein	*Prevotella ruminicola* 23 YP_003574497 ($6e^{-35}$)	68/96 (71%)
17	165	Hypothetical protein	*Prevotella ruminicola* 23 YP_003574496 ($3e^{-63}$)	116/147 (79%)
18	773	Hypothetical protein	*Prevotella ruminicola* 23 YP_003574495 (0.0)	525/810 (65%)
19	145	Structural protein P5	*Prevotella ruminicola* 23 YP_003574013 ($4e^{-53}$)	93/140 (66%)
20	360	DNA polymerase IV	*Prevotella ruminicola* 23 YP_003573872 (0.0)	323/367 (88%)
21	273	Vancomycin B-type resistance protein VanW	*Ruminococcus albus* 7 ($6e^{-103}$)	176/273 (64%)
22	468	Membrane protein	*Bacteroidetes oral* taxon 274 str. F0058 ($4e^{-131}$)	232/461 (50%)
23	154	Transcription elongation factor GreA	*Prevotella ruminicola* 23 YP_003573861 ($1e^{-68}$)	126/154 (82%)
24	238	RNA polymerase sigma factor RpoD	*Prevotella ruminicola* 23 YP_003574537 ($1e^{-95}$)	172/235 (73%)
25	194	Protein co-occurring with transport systems (COG1739)	*Prevotella ruminicola* 23 YP_003574362 ($7e^{-99}$)	171/194 (88%)
26	1086	Carbamoyl-phosphate synthase large chain	*Prevotella ruminicola* 23 YP_003574361 (0.0)	1043/1094 (95%)
27	826	ATP-dependent DNA helicase UvrD/PcrA	*Prevotella ruminicola* 23 YP_003574330 (0.0)	685/846 (81%)
28	43	Hypothetical protein	*Prevotella ruminicola* 23 YP_003574329 ($2e^{-13}$)	35/43 (81%)
29	482	Lipoprotein	*Prevotella ruminicola* 23 YP_003574328 ($2e^{-145}$)	262/487 (54%)
30	270	Similar to glycogen synthase	*Prevotella ruminicola* 23 YP_003574327 ($2e^{-117}$)	202/269 (75%)

Table S8 –continued

ORF	Protein size (aa)	Putative function	Most similar homolog (e-value)	Identity (overlapped aa)
31	168	Transcriptional regulator, AraC/XylS family	*Prevotella ruminicola* 23 YP_003574325 ($1e^{-72}$)	131/154 (85%)
32	656	Dipeptidyl-peptidase III	*Prevotella ruminicola* 23 YP_003574324 (0.0)	506/647 (78%)
33	206	Peptidase, M49 family	*Prevotella ruminicola* 23 YP_003574323 ($4e^{-81}$)	147/198 (74%)
34	165	Ferric uptake regulation protein FUR	*Prevotella ruminicola* 23 YP_003574322 ($8e^{-68}$)	120/144 (83%)
35	423	Adenylosuccinate synthetase	*Prevotella ruminicola* 23 YP_003574321 (0.0)	400/422 (95%)
36	453	Histidyl-tRNA synthetase	*Prevotella ruminicola* 23 YP_003574319 (0.0)	413/450 (92%)
**37**	**281**	**Rhamnogalacturonan acetylesterase**	***Prevotella ruminicola* 23 YP_003574318 ($1e^{-119}$)**	**198/254 (78%)**
38	184	Rubrerythrin	*Prevotella ruminicola* 23 YP_003574317 ($2e^{-96}$)	172/183 (94%)
39	327	Putative lipoprotein	*Prevotella ruminicola* 23 YP_003574316 ($9e^{-115}$)	195/324 (60%)

ORFs are numbered following the sense 5’-3’.

Table S9 Predicted protein-coding genes in fosmid LAB8M16

ORF	Protein size (aa)	Putative function	Most similar homolog (e-value)	Identity (overlapped aa)
1	172	Transposase	*Bacteroides* sp. ($6e^{-64}$)	121/152 (80%)
2	79	Hypothetical protein	None	
3	61	Hypothetical protein	*Prevotella copri* DSM 18205 ($1e^{-21}$)	45/61 (74%)
4	762	Alpha-xylosidase	*Prevotella ruminicola* 23 YP_003575493 (0.0)	631/742 (85%)
5	671	Excinuclease ABC subunit B	*Prevotella ruminicola* 23 YP_003574669 (0.0)	555/676 (82%)
6	275	Lipoprotein protein, putative	*Prevotella ruminicola* 23 YP_003574668 ($8e^{-102}$)	180/275 (65%)
7	112	Hypothetical protein	*Prevotella ruminicola* 23 YP_003574667 ($6e^{-53}$)	101/112 (90%)
8	116	Lipoprotein	*Prevotella buccae* ATCC 33574 ($7e^{-14}$)	59/153 (39%)
9	215	Pyruvate formate-lyase activating enzyme	*Prevotella ruminicola* 23 YP_003574559 ($7e^{-107}$)	183/212 (86%)
10	748	Pyruvate formate-lyase	*Prevotella ruminicola* 23 YP_003574558 (0.0)	737/748 (99%)
11	1190	Pyruvate-flavodoxin oxidoreductase	*Prevotella ruminicola* 23 YP_003574550 (0.0)	1124/1192 (94%)
12	48	Hypothetical protein	None	
13	1259	Beta-galactosidase	*Bacteroides* sp. 20_3 (0.0)	690/1261 (55%)
14	37	Hypothetical protein	None	
15	550	Beta-xylosidase	*Bacteroides egghertii* DSM 20697 (0.0)	316/553 (57%)
16	823	Hypothetical protein	*Bacteroides egghertii* DSM 20697 (0.0)	383/684 (56%)
17	434	HipA domain-containing protein	*Prevotella ruminicola* 23 YP_003575672 ($8e^{-168}$)	277/427 (65%)
18	40	Hypothetical protein	*Chlorobium phaeobacteroides* BS1 ($4e^{-06}$)	28/39 (72%)
19	460	GTPase and tRNA-U34 5-formylation enzyme TrmE	*Prevotella ruminicola* 23 YP_003574055 (0.0)	332/458 (72%)
20	291	Purine nucleoside phosphorylase	*Prevotella ruminicola* 23 YP_003574056 ($6e^{-149}$)	248/289 (86%)
21	215	Hypothetical protein	*Bacteroides coprophilus* DSM 18228 ($7e^{-40}$)	82/207 (40%)
22	170	Hypothetical protein	*Paraprevotella xylaniphila* YIT 11841 ($3e^{-20}$)	63/190 (33%)
23	248	tRNA(Cytosine32)-2-thiocytidine synthetase	*Prevotella ruminicola* 23 YP_003574058 ($3e^{-88}$)	154/247 (62%)
24	107	Membrane protein, putative	*Prevotella copri* DSM 18205 ($4e^{-43}$)	84/106 (79%)
25	429	Glutamate synthase [NADPH] small chain	*Prevotella ruminicola* 23 YP_003574059 (0.0)	399/427 (93%)

ORFs are numbered following the sense 5'-3'. ORF 1 was situated on contig00001, ORFs 2-25 on contig00003.

Table S10 Predicted protein-coding genes in fosmid LAB9D24

ORF	Protein size (aa)	Putative function	Most similar homolog (e-value)	Identity (overlapped aa)
1	249	Ferredoxin	*Clostridium hiranonis* DSM 13275 ($4e^{-55}$)	103/244 (42%)
2	221	Hypothetical protein	*Ruminococcus flavefaciens* FD-1 ($5e^{-07}$)	58/219 (44%)
3	281	NAD dependant epimerase/dehydratase	*Butyrivibrio proteoclasticus* B316 ($2e^{-126}$)	221/282 (78%)
4	593	ABC transporter, substrate-binding protein	*Butyrivibrio fibrisolvens* 16/4 ($3e^{-152}$)	275/522 (53%)
5	75	Hypothetical protein	None	
6	104	Hypothetical protein	None	
7	88	Hypothetical protein	None	
8	610	Hypothetical protein	None	
9	360	Hypothetical protein	None	
10	384	Hypothetical protein	None	
11	435	Diguanylate cyclase	*Ruminococcus flavefaciens* FD-1 ($2e^{-131}$)	223/413 (54%)
12	268	Hypothetical protein	*Ruminococcus albus* 7 ($1e^{-88}$)	156/262 (60%)
13	640	Diguanylate cyclase	*Roseburia intestinalis* XB6B4 ($2e^{-67}$)	186/644 (29%)
14	243	Hypothetical protein	None	
15	125	Hypothetical protein	None	
16	268	Hypothetical protein	None	
17	1565	Hypothetical protein	*Haliscomenobacter hydrossis* DSM 1100 ($2e^{-84}$)	385/1637 (24%)
18	275	Ribosomal protein L11 methyltransferase	*Bacteroides eggerthii* 1_2_48FAA ($3e^{-66}$)	123/278 (44%)
19	248	Triosephosphate isomerase (EC 5.3.1.1)	*Sphingobacterium* sp. 21 ($1e^{-69}$)	123/239 (51%)
20	252	Hypothetical protein	*Prevotella disiens* FB035-09AN ($2e^{-33}$)	83/248 (33%)
21	79	Peptidase M23	*Fluviicola taffensis* DSM 16823 ($5e^{-04}$)	20/47 (43%)
22	107	Xyloside transporter XynT	*Prevotella ruminicola* 23 ($8e^{-27}$)	60/77 (78%)
23	490	Hypothetical protein	None	
24	122	Hypothetical protein	*Ruminococcus torques* ATCC 27756 ($5e^{-07}$)	26/59 (44%)
25	348	DNA polymerase III beta subunit	*Flavobacterium johnsoniae* UW101 ($1e^{-115}$)	200/349 (57%)
26	253	Hypothetical protein	None	
27	207	Hypothetical protein	None	
28	339	Phosphoenolpyruvate carboxykinase [ATP]	*Bacteroides vulgatus* ATCC 8482 ($1e^{-56}$)	108/133 (81%)
29	298	Hypothetical protein	None	
30	277	Hypothetical protein	None	
31	39	Hypothetical protein	None	

ORFs are numbered following the sense 5'-3'. ORFs 1-14 was situated on contig00001, ORFs 15-16 on contig00002, ORFs 17-22 on contig00005, ORFs 23-24 on contig00006, ORFs 25-31 on contig00007.

Table S11 Predicted protein-coding genes in fosmid LAB9P23

ORF	Protein size (aa)	Putative function	Most similar homolog (e-value)	Identity (overlapped aa)
1	354	Hypothetical protein	*Prevotella ruminicola* 23 YP_003574374 ($2e^{-167}$)	275/350 (79%)
2	115	Putative anti-sigma regulatory factor	*Bacteroides salanitronis* DSM18170 ($2e^{-21}$)	48/88 (55%)
3	93	Putative anti-anti sigma factor	*Bacteroides fragilis* 3_1_12 ($6e^{-18}$)	43/87 (49%)
4	381	Neopullulanase	*Streptococcus* sp. M334 ($9e^{-36}$)	120/404 (30%)
5	645	Serine phosphatase RsbU, regulator of sigma subunit	*Prevotella ruminicola* 23 YP_003574202 ($1e^{-126}$)	268/676 (40%)
**6**	**352**	**Esterase**	**uncultured bacterium ABI17943 ($1e^{-155}$)**	**265/366 (72%)**
7	415	Outer membrane efflux protein	*Prevotella melaninogenica* D18 ZP_06409119 ($1e^{-97}$)	186/383 (49%)
8	1065	Acriflavin resistance protein	*Bacteroides cellulosilyticus* DSM 14838 (0.0)	604/1046 (58%)
9	335	Probable Co/Zn/Cd efflux system membrane fusion protein	*Bacteroides coprophilus* DSM 18228 ($2e^{-87}$)	157/324 (48%)
10	182	Chloramphenicol O-acetyltransferase	*Prevotella ruminicola* 23 YP_003575710 ($2e^{-67}$)	115/182 (63%)
11	418	Aminopeptidase	*Cytophaga hutchinsonii* ATCC 33406 ($4e^{-54}$)	134/400 (34%)
12	184	Conserved hypothetical protein, putative transport protein	*Prevotella ruminicola* 23 YP_003575836 ($6e^{-89}$)	158/184 (86%)
13	121	Conserved hypothetical protein, putative transport protein	*Prevotella ruminicola* 23 YP_003575836 ($1e^{-53}$)	
14	249	Hypothetical protein	*Prevotella ruminicola* 23 YP_003574400 ($1e^{-53}$)	101/120 (84%)
15	487	Indolepyruvate oxidoreductase subunit IorA	*Prevotella ruminicola* 23 YP_003574357 (0.0)	436/471 (93%)
16	190	Indolepyruvate oxidoreductase subunit IorB	*Prevotella ruminicola* 23 YP_003574356 ($2e^{-97}$)	179/190 (94%)
17	436	Aspartate aminotransferase	*Prevotella ruminicola* 23 YP_003574355 (0.0)	400/436 (92%)
18	458	Transposase	*Bacteroides* sp. 4_3_47FAA (0.0)	360/457 (79%)
19	251	Transposase, IS4 family	*Prevotella buccalis* ATCC 35310 ($2e^{-104}$)	179/240 (75%)
20	50	Hypothetical protein	None	
21	434	Protein tyrosine/serine phosphatase	*Lactobacillus salivarius* ATCC 11741 ($9e^{-10}$)	34/93 (37%)
22	116	Hypothetical protein	*Prevotella oralis* ATCC 33269 ($5e^{-25}$)	57/116 (49%)
23	305	Two-component system sensor histidine kinase	*Prevotella ruminicola* 23 YP_003575656 ($4e^{-84}$)	161/265 (61%)
24	141	Biotin carboxyl carrier protein of methylmalonyl-CoA decarboxylase	*Prevotella ruminicola* 23 YP_003574547 ($5e^{-45}$)	101/142 (71%)
25	49	Hypothetical protein	*Prevotella* sp. *oral* taxon 299 str. F0039 ($5e^{-10}$)	31/45 (69%)
26	521	Methylmalonyl-CoA decarboxylase, alpha chain	*Prevotella ruminicola* 23 YP_003574546 (0.0)	502/521 (96%)
27	136	Methylmalonyl-CoA epimerase	*Prevotella ruminicola* 23 YP_003574545 ($1e^{-71}$)	134/149 (99%)
28	785	Putative ferric aerobactin receptor	*Prevotella ruminicola* 23 YP_003574543 (0.0)	576/755 (76%)
29	1281	5'-nucleotidase	*Prevotella ruminicola* 23 YP_003575483 (0.0)	599/789 (76%)

Table S11 –continued

ORF	Protein size (aa)	Putative function	Most similar homolog (e-value)	Identity (overlapped aa)
30	347	Endonuclease I	*Paraprevotella xylaniphila* YIT 11841 ($4e^{-84}$)	159/287 (55%)
31	227	TPR-repeat-containing protein	*Bacteroides cellulosilyticus* DSM 14838 ($3e^{-24}$)	75/216 (35%)
32	139	Putative activity regulator of membrane protease YbbK	*Prevotella ruminicola* 23 YP_003574541 ($5e^{-47}$)	101/135 (75%)
33	317	Putative stomatin/prohibitin-family membrane protease subunit YbbK	*Prevotella ruminicola* 23 YP_003574540 ($2e^{-176}$)	303/317 (96%)
34	295	Dihydrodipicolinate synthase	*Prevotella ruminicola* 23 YP_003574539 ($9e^{-160}$)	273/295 (93%)
35	295	Transcriptional regulator, AraC family	*Prevotella ruminicola* 23 YP_003574538 ($6e^{-143}$)	237/294 (81%)
36	385	Putative tetratricopeptide repeat family protein	*Prevotella ruminicola* 23 YP_003574401 ($2e^{-130}$)	225/377 (60%)

ORFs are numbered following the sense 5’-3’. ORF 1 was situated on contig00002, ORFs 2-9 on contig00003, ORFs 10-14 on contig00004, ORFs 15-21 on contig00006, ORFs 22-36 on contig00010.

Table S12 Best matches obtained using BLASTN for the lipolytic genes

Fosmid	Gene	Best hit (accession number)	Nucleotide identities (%)	E-value
SAB5A16	*lip1*	Uncultured prokaryote o23 gene for ester hydrolase (AJ811969)	1229/1589 (77%)	0.0
	lip2		589/758 (78%)	$3e^{-169}$
	lip3		646/867 (75%)	$9e^{-158}$
SAB16A18	*lip4*		675/912 (74%)	$2e^{-160}$
	lip5		608/758 (80%)	0.0
	lip6		1172/1546 (76%)	0.0
SAB16E6	*lip7*	Uncultured bacterium Est5S (est5S) gene (DQ788540)	776/1103 (70%)	$4e^{-134}$
	lip8	Unidentified microorganism phagemid clone pBKR.43 (AM050333)	660/880 (75%)	$3e^{-161}$
	pl1	None		
SAB18J4	*lip9*	Uncultured prokaryote o23 gene for ester hydrolase (AJ811969)	1075/1565 (69%)	$1e^{-156}$
	lip10		1327/1661 (80%)	0.0
SAB28M4	*lip11*	Unidentified microorganism phagemid clone pBKR.43 (AM050333)	660/880 (75%)	$3e^{-161}$
	lip12	Uncultured bacterium Est5S (est5S) gene (DQ788540)	779/1104 (71%)	$7e^{-138}$
	pl2	None		
LAB4P4	*lip13*	Lipase/acylhydrolase from *Prevotella ruminicola* 23 (CP002006)	528/737 (72%)	$8e^{-104}$
LAB9P23	*lip14*	Uncultured bacterium Est5S (est5S) gene (DQ788540)	838/1109 (76%)	0.0

Table S13 Best matches using BLASTP for the amino acid sequences deduced from the lipolytic genes

Fosmid	Protein	Best hits (accession number)	Amino acid identities (%)	E-value
SAB5A16	lip1	Ester hydrolase from uncultured prokaryote (CAH19079)	386/515 (75%)	0.0
	lip2		326/518 (63%)	0.0
	lip3	Esterase EstZ3 from uncultured bacterium (ADE28720)	187/310 (60%)	$2e^{-123}$
		Esterase EstGK1 from uncultured bacterium (ADE28719)	176/318 (55%)	$3e^{-119}$
SAB16A18	lip4	Esterase EstZ3 from uncultured bacterium (ADE28720)	187/310 (60%)	$4e^{-123}$
		Esterase EstGK1 from uncultured bacterium (ADE28719)	176/318 (55%)	$7e^{-120}$
	lip5	Ester hydrolase from uncultured prokaryote (CAH19079)	327/518 (63%)	0.0
	lip6		387/515 (75%)	0.0
SAB16E6	lip7	Esterase 5S from uncultured bacterium (ABI17943)	232/365 (64%)	$1e^{-171}$
	lip8	Putative esterase/lipase from unidentified microorganism (CAJ19128)	218/280 (78%)	$4e^{-161}$
		α/β hydrolase from *Fibrobacter succinogenes* S85 (YP_003248122)	142/247 (57%)	$4e^{-106}$
	pl1	Patatin family phospholipase from *Prevotella oralis* ATCC33269 (ZP_08085180)	167/416 (40%)	$6e^{-105}$
SAB18J4	lip9	Ester hydrolase from uncultured prokaryote (CAH19079)	324/517 (63%)	0.0
	lip10		433/515 (84%)	0.0
SAB28M4	lip11	Putative esterase/lipase from unidentified microorganism (CAJ19128)	238/315 (76%)	$1e^{-179}$
	lip12	Esterase 5S from uncultured bacterium (ABI17943)	232/365 (64%)	$9e^{-174}$
	pl2	Patatin family phospholipase from *Prevotella oralis* ATCC33269 (ZP_08085180)	371/732 (51%)	0.0
LAB4P4	lip13	Lipase/acylhydrolase from *Prevotella ruminicola* 23 (YP_003574318)	198/254 (78%)	$6e^{-153}$
		Putative pectinesterase from *Prevotella copri* DSM18205 (EFB34556)	160/271 (59%)	$7e^{-115}$
LAB9P23	lip14	Esterase 5S from uncultured bacterium (ABI17943)	265/366 (72%)	0.0

Figure S1 Example of positive primary screening on spirit blue agar after 48h growth. The arrow shows clone SAB5A16 appearing positive, i.e. blue against the clear background.

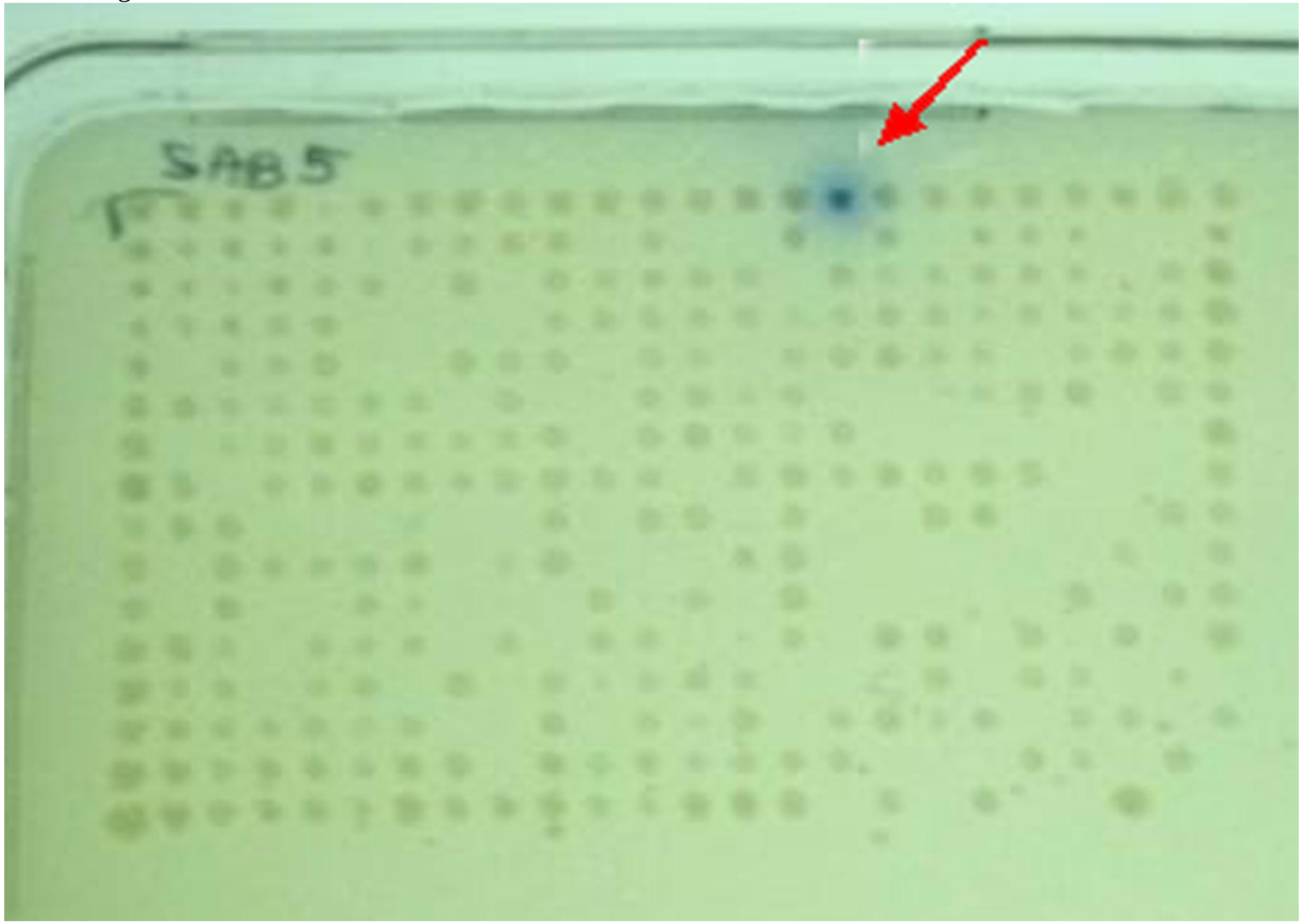

Figure S2 Multiple amino acids alignments of lip8, lip11 and lipolytic enzymes from the HSL family. Alignment of Pfam conserved domains. The accession numbers of the aligned sequences are for the following organisms: ZP_00943646, esterase from *Ralstonia solanacearum* UW551; YP_442879, lipase/esterase from *Burkholderia thailandensis* E264; AAC38151, lipase from *Pseudomonas* sp. B11-1; AAB89533, carboxylesterase estA from *Archaeoglobus fulgidus* DSM 3404; AAC41424, lipase-like enzyme from *Cupriavidus necator*. Conserved motifs characteristic of lipolytic enzymes classified in family IV are highlighted. The possible catalytic triad (Serine (S), Aspartic acid (D) or Glutamic acid (E), Histidine (H)) is shown at the top of the alignment whenever necessary.

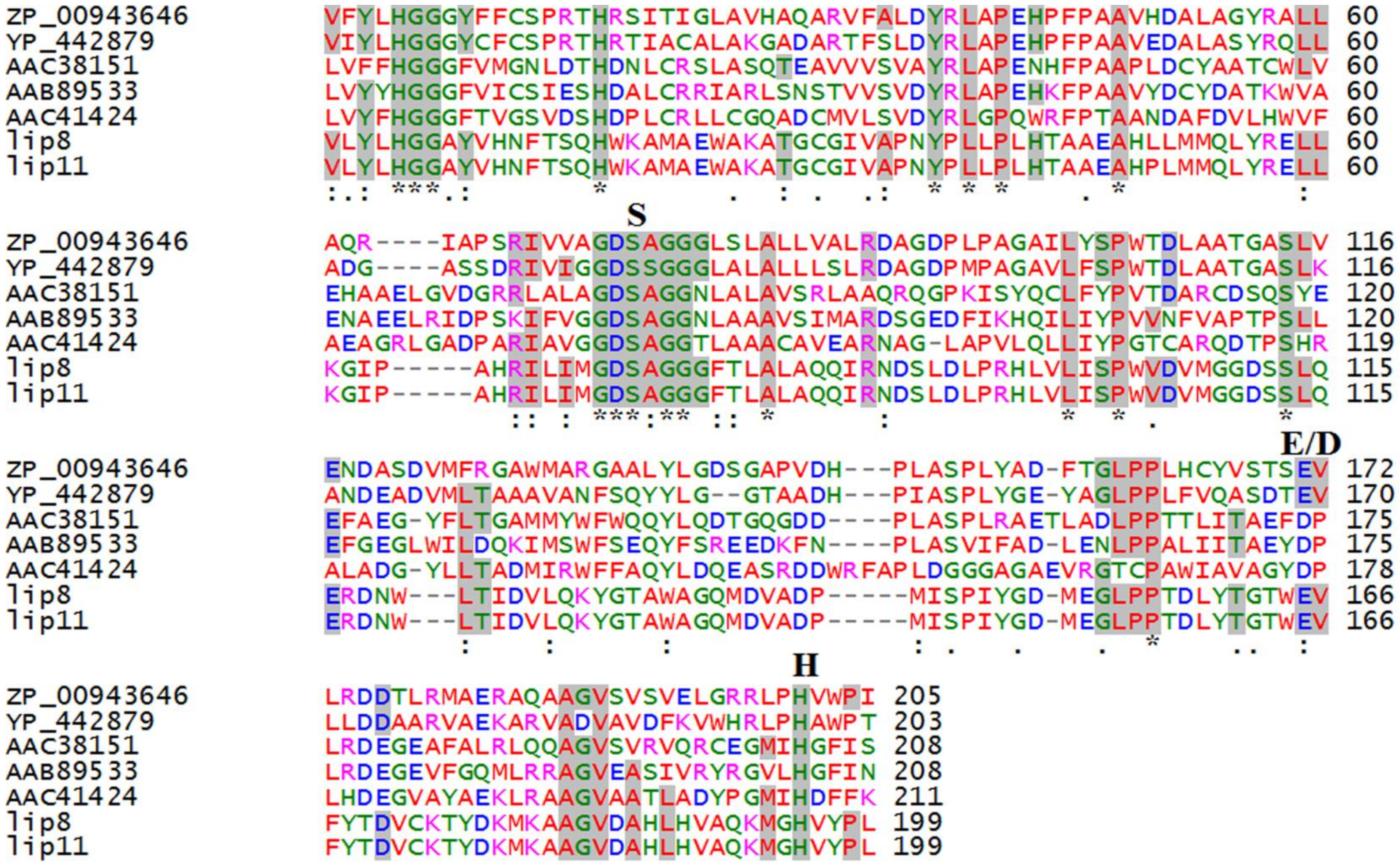

Figure S3 Conserved motifs from multiple amino acids alignments of lip1, lip2, lip5, lip6, lip9 and lip10 and lipolytic enzymes from family VII. Alignment of Pfam conserved domains. The accession numbers of the aligned sequences are for the following organisms: Q01470, phenylcarbamate hydrolase from *Arthrobacter oxydans*; CAA22794, putative carboxylesterase from *Streptomyces coelicolor* A3(2); YP_001069337, carboxylesterase from *Mycobacterium* sp. JLS; YP_001136305, carboxylesterase from *Mycobacterium gilvum* PYR-GCK; ZP_03881667, carboxylesterase from *Haliangium ochraceum*; BAA76305, polyurethane esterase from *Delftia acidovorans*; AAL82802, paraben-hydrolysing esterase precursor from *Enterobacter cloacae*; ZP_03620470, carboxylesterase from *Tolumonas auensis* DSM 9187; ZP_04761385, carboxylesterase from *Acidovorax delafieldii* 2AN; P37967, para-nitrobenzyl esterase from *Bacillus subtilis*. Conserved motifs are highlighted. The possible catalytic triad (Serine (S), Aspartic acid (D) or Glutamic acid (E), Histidine (H)) is shown at the top of the alignment whenever necessary.

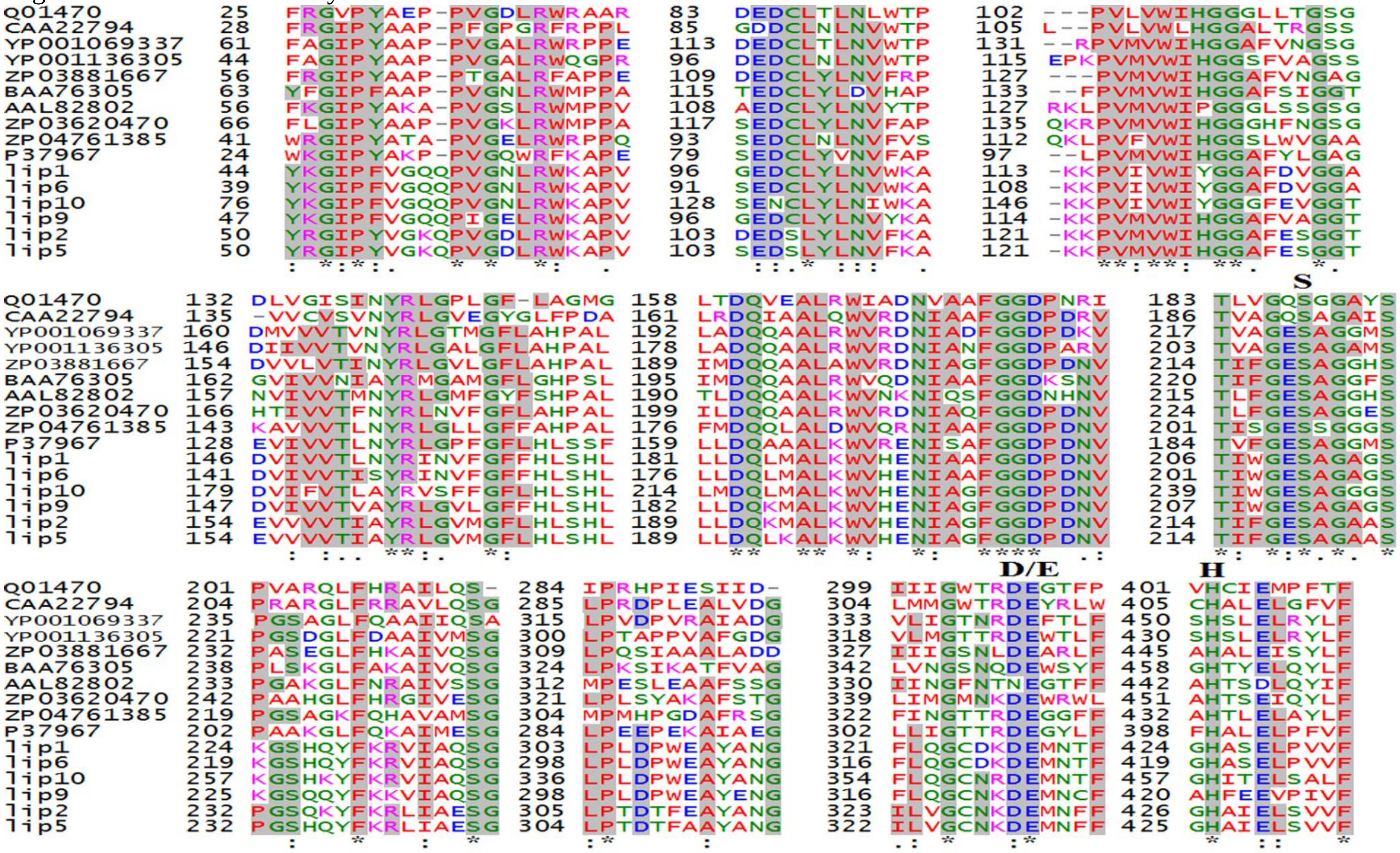

Figure S4 Multiple alignment of lip13 and lipolytic enzymes from the GDSL family. Alignment of Pfam conserved domains. The accession numbers of the aligned sequences are for the following organisms: CAA47020, triacylglycerol lipase from *Photorhabdus luminescens*; AAC38796, outer membrane esterase from *Salmonella typhimurium*; AAB61674, lipase/esterase from *Pseudomonas aeruginosa* PAO1. Conserved motifs are highlighted. The possible catalytic triad (Serine (S), Aspartic acid (D), Histidine (H)) is shown at the top of the alignment whenever necessary.

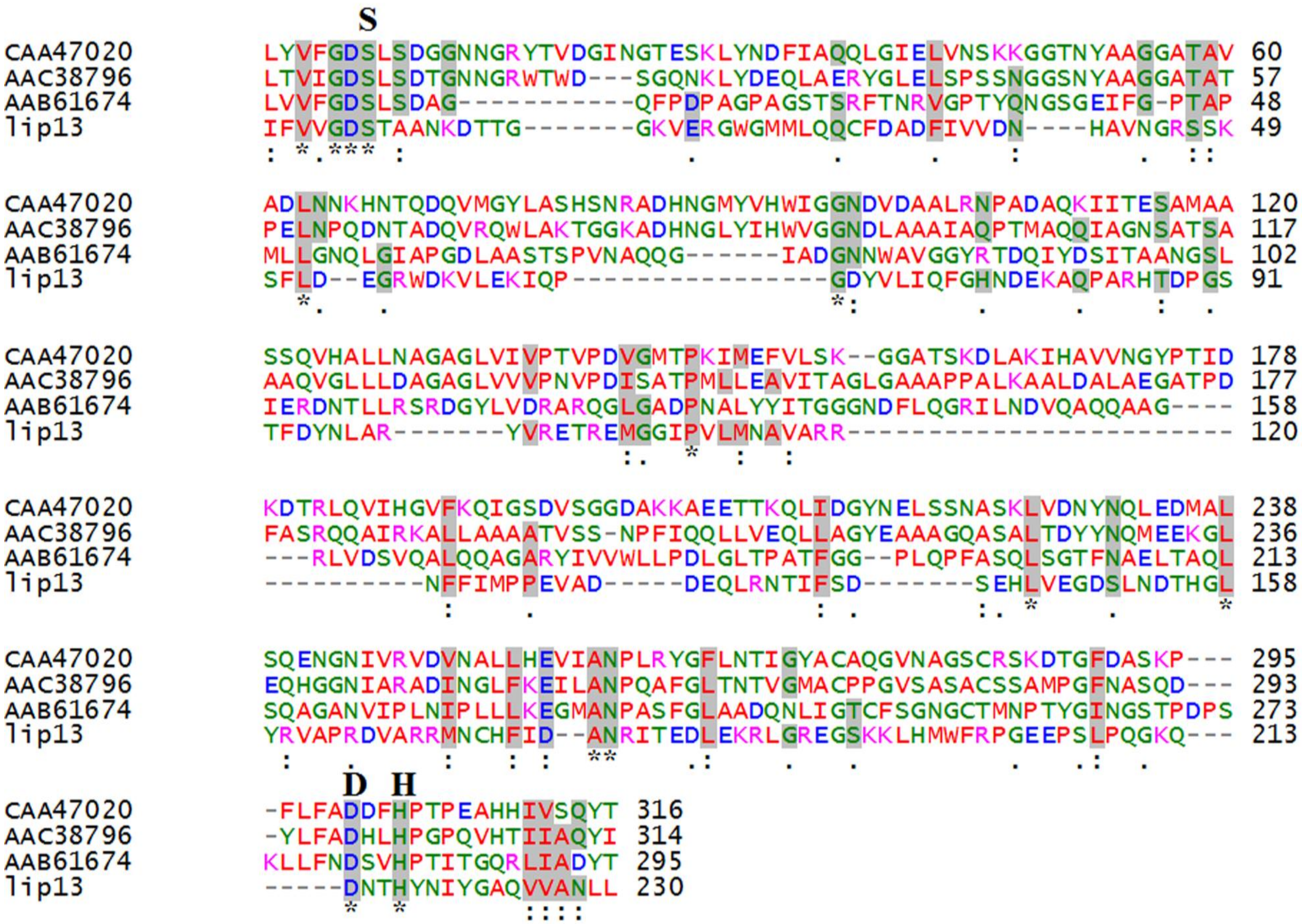

Figure S5 Multiple amino acids alignments of lip3, lip4, lip7, lip12, lip14 and lipolytic enzymes from the subfamily I.7. The accession numbers of the aligned sequences are for the following organisms: Q8NU59, Q8NU60, predicted triacylglycerol hydrolases from *Corynebacterium glutamicum*; AAB71210, lipase LipA from *Streptomyces cinnamoneus*; CAA67627, triacylglycerol lipase from *Propionibacterium acnes*. Conserved motifs are highlighted. The possible catalytic triad (Serine (S), Aspartic acid (D), Histidine (H)) is shown at the top of the alignment whenever necessary.

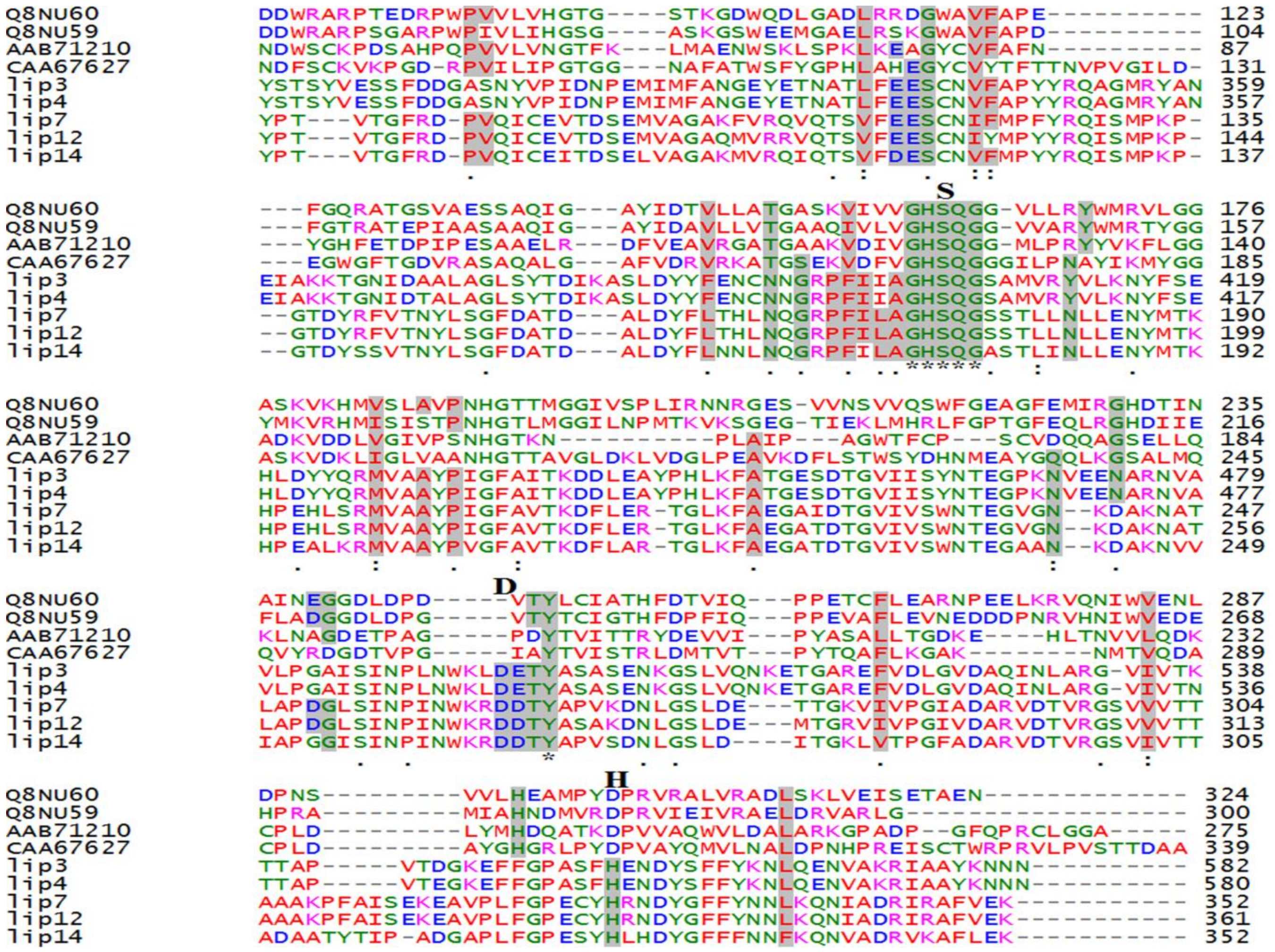

Supplement: Supplementary file 1 — (PDF 2509 kb) [file 253_2014_6355_MOESM1_ESM.pdf]
